# Neuropathy-related mutations alter the membrane binding properties of the human myelin protein P0 cytoplasmic tail

**DOI:** 10.1371/journal.pone.0216833

**Published:** 2019-06-07

**Authors:** Arne Raasakka, Salla Ruskamo, Robert Barker, Oda C. Krokengen, Guro H. Vatne, Cecilie K. Kristiansen, Erik I. Hallin, Maximilian W. A. Skoda, Ulrich Bergmann, Hanna Wacklin-Knecht, Nykola C. Jones, Søren V. Hoffmann, Petri Kursula

**Affiliations:** 1 Department of Biomedicine, University of Bergen, Bergen, Norway; 2 Faculty of Biochemistry and Molecular Medicine, University of Oulu, Oulu, Finland; 3 Biocenter Oulu, University of Oulu, Oulu, Finland; 4 School of Physical Sciences, University of Kent, Kent, United Kingdom; 5 ISIS Neutron and Muon Source, Science & Technology Facilities Council, Rutherford Appleton Laboratory, Didcot, United Kingdom; 6 Division of Physical Chemistry, Department of Chemistry, Lund University, Lund, Sweden; 7 European Spallation Source ERIC, Lund, Sweden; 8 ISA, Department of Physics and Astronomy, Aarhus University, Aarhus C, Denmark; Martin-Luther-Universitat Halle-Wittenberg, GERMANY

## Abstract

Schwann cells myelinate selected axons in the peripheral nervous system (PNS) and contribute to fast saltatory conduction *via* the formation of compact myelin, in which water is excluded from between tightly adhered lipid bilayers. Peripheral neuropathies, such as Charcot-Marie-Tooth disease (CMT) and Dejerine-Sottas syndrome (DSS), are incurable demyelinating conditions that result in pain, decrease in muscle mass, and functional impairment. Many Schwann cell proteins, which are directly involved in the stability of compact myelin or its development, are subject to mutations linked to these neuropathies. The most abundant PNS myelin protein is protein zero (P0); point mutations in this transmembrane protein cause CMT subtype 1B and DSS. P0 tethers apposing lipid bilayers together through its extracellular immunoglobulin-like domain. Additionally, P0 contains a cytoplasmic tail (P0ct), which is membrane-associated and contributes to the physical properties of the lipid membrane. Six CMT- and DSS-associated missense mutations have been reported in P0ct. We generated recombinant disease mutant variants of P0ct and characterized them using biophysical methods. Compared to wild-type P0ct, some mutants have negligible differences in function and folding, while others highlight functionally important amino acids within P0ct. For example, the D224Y variant of P0ct induced tight membrane multilayer stacking. Our results show a putative molecular basis for the hypermyelinating phenotype observed in patients with this particular mutation and provide overall information on the effects of disease-linked mutations in a flexible, membrane-binding protein segment. Using neutron reflectometry, we additionally show that P0ct embeds deep into a lipid bilayer, explaining the observed effects of P0ct on the physical properties of the membrane.

## Introduction

Fast saltatory nerve impulse conduction requires myelin, a structure composed of tightly stacked lipid bilayers that wrap around selected axonal segments in the central and peripheral nervous systems (CNS and PNS, respectively). The insulative nature of myelin enables efficient nerve impulse propagation, and the destruction of myelin, demyelination, underlies a range of chronic diseases. In the PNS, peripheral neuropathies affect Schwann cell compact myelin. These include Charcot-Marie-Tooth disease (CMT) and its more severe, rapidly progressive form known as Dejerine-Sottas syndrome (DSS), which cause incurable chronic disability [1,2]. CMT and DSS manifest through both dominant and recessive inheritance, and they harbour a strong genetic component, typically caused by mutations in proteins relevant for the formation and stability of PNS myelin, while axonal forms also exist.

Myelin protein zero (P0) is a type I transmembrane protein consisting of an extracellular immunoglobulin (Ig)-like domain [3], a single transmembrane helix, and a 69-residue C-terminal cytoplasmic tail (P0ct). P0ct is likely to be involved in the regulation of myelin membrane behaviour, supporting the arrangement of the P0 Ig-like domains in the extracellular space upon the formation of the myelin intraperiod line [4–6]. P0ct contains a neuritogenic segment, which can be used to induce experimental autoimmune neuritis (EAN) in animal models [7]. *In vitro*, P0ct is disordered in aqueous solution, gaining secondary structure upon binding to negatively charged phospholipids [4,5]. In its lipid-bound state, P0ct affects the phase behaviour of lipids and promotes the fusion of lipid vesicles. High-degree molecular order, most likely from stacked lipid bilayers, can be detected *via* X-ray diffraction of P0ct-bound membranes [5]. This suggests that P0ct harbours a structural role in mature myelin.

Dozens of mutations have been identified in P0, most of which affect the Ig-like domain. These mutations affect myelin morphology and integrity, leading to the development of peripheral neuropathies [8,9]. Six known missense mutations are located within P0ct, of which four cause dominant demyelinating CMT type 1B (CMT1B). These include T216ER [10], D224Y (also referred to as D195Y and D234Y) [11–13], R227S [9], and the deletion of Lys236 (K236del) [14]. In addition, K236E has been linked to dominant axonal CMT type 2I (CMT2I) [15], and A221T, which was discovered as a co-mutation together with the deletion of Val42 in the Ig-like domain, was identified in a patient with DSS [16]. How these mutations relate to CMT/DSS etiology is not known, although P0 mutations have been linked to the unfolded protein response (UPR) [17–19], indicating issues in either translation or folding that induce stress within the endoplasmic reticulum (ER).

Considering the small size of P0ct and the nature of the disease mutations in it, many of which change its electrostatic charge, impairment in the function of P0ct as a membrane binding/stabilizing segment is a possible functional mechanism. We used methodologies established earlier for myelin basic protein (MBP) [20] and wild-type P0ct (wt-P0ct) [5,21] to characterize structure-function relationships of the CMT- and DSS-related P0ct variants. Our results suggest that D224Y is a hypermyelinating gain-of-function mutation, which is in line with the clinically relevant phenotype of abnormally thick myelin sheaths [11].

## Results

We earlier studied the binding of MBP and P0ct to model lipid membranes [5,20,21], using a biophysical workflow that allows the determination of binding affinity, level of folding, alteration of lipid phase behaviour, quantification and visualization of vesicle aggregation and fusion, and supported lipid bilayer (SLB) stacking. In the current study, we examined whether and how CMT and DSS mutations within P0ct influence its structure and function. For this purpose, we expressed and purified the wild-type protein and six mutant variants, each harbouring one of the following amino acid changes: T216ER, A221T, D224Y, R227S, K236E, and K236del.

### Characterization of P0ct CMT mutants

**Fig 1:**
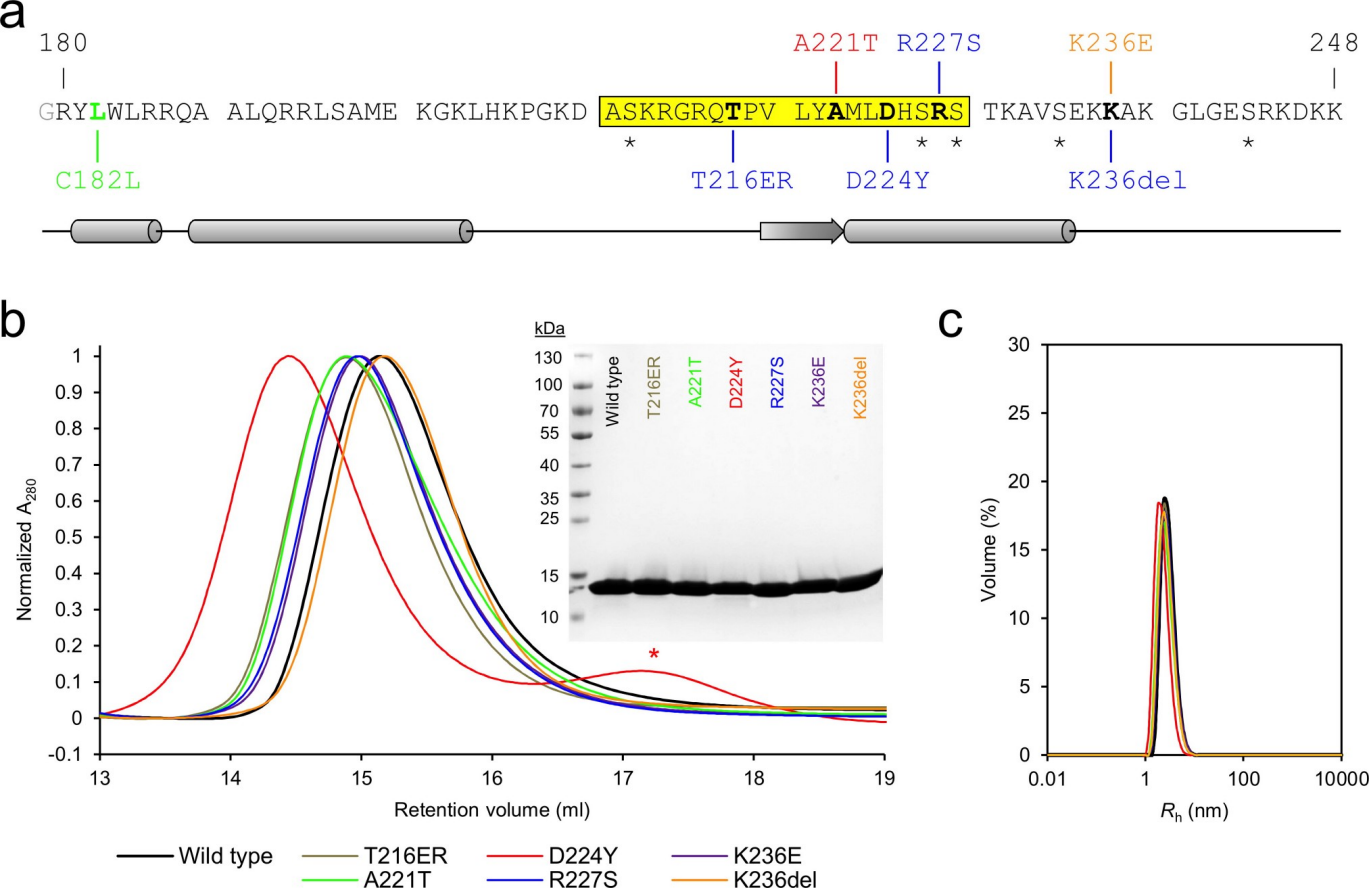
Overview of P0ct mutations. (a) The sequence of the wt-P0ct construct, corresponding to amino acids 180–248 of the human P0 precursor, with an extra N-terminal Gly residue (gray) left behind from affinity tag cleavage. The Cys182 palmitoylation site was mutated into a Leu (green) in all constructs. Putative serine phosphorylation sites are indicated with asterisks. Residues affected by disease mutations are in bold. CMT1B, CMT2I, and DSS point mutations are shown in blue, red, and orange, respectively. The sequence highlighted in yellow corresponds to the neuritogenic segment used in EAN models [7]. Secondary structure prediction is shown below. (b) SEC traces of wt-P0ct and mutants as determined using a Superdex 75 10/300GL column. Note the slightly lower retention volume of D224Y, for which the chromatography had to be performed with a different running buffer than for the other variants. The degradation products (red asterisk) present with D224Y could be completely removed using SEC. The final purity of each P0ct variant (4 μg per lane) as determined using SDS-PAGE is shown as inset. (c) DLS data of P0ct variants display good monodispersity with minimal variation in *R*_h_.

wt-P0ct and the six CMT variants were purified to homogeneity. Most mutants were straightforward to purify, showing identical behaviour to wt-P0ct in size-exclusion chromatography (SEC) (Fig 1B). D224Y, on the other hand, had to be gel filtered at a higher pH and salt concentration than the others, and while yields were generally lower, minor amounts of degradation were present and the migration in SEC was altered, albeit not in denaturing gel electrophoresis (SDS-PAGE) (Fig 1B. S1 Fig). In dynamic light scattering (DLS), all variants displayed a similar hydrodynamic radius (*R*_h_) and an absence of aggregation (Fig 1C, S1 Table). All of the variants showed high apparent molecular weight in SDS-PAGE, which reflects the intrinsically disordered nature of P0ct [5]. The molecular weight and the presence of the mutations were confirmed using mass spectrometry (Table 1). The total yields of the purified mutant proteins were different from wt-P0ct (S1 Fig, Table 1), most mutants giving larger yields, with the exception of D224Y. It should be noted that all mutants were expressed as maltose-binding protein fusions. Thus, mutations, which represent small changes in the overall sequence and size of the fusion protein, can affect the expression and purification behaviour.

**Table 1:** Recombinant protein characterization.

			Purification		Molecular weight determination			Peptide fingerprinting
Variant*	Condition	pI**	Yield***	Solubility	Measured	Theoretical**	Difference	Mutation confirmed
wt-P0ct	-	11.11	2.1 ± 0.4	++	7989.0	7990.35	-1.35	-
T216ER	CMT1	11.08	4.2 ± 0.4	+++	8173.0	8174.54	-1.54	yes
A221T	DSS	11.11	5.0 ± 0.7	+++	8018.0	8020.37	-2.37	yes
D224Y	CMT1	11.12	0.8 ± 0.3	+	8037.0	8038.43	-1.43	yes
R227S	CMT1	10.89	6.1 ± 2.0	+++	7919.0	7921.24	-2.24	yes
K236E	CMT2	10.85	5.1 ± 1.8	+++	7989.0	7991.29	-2.29	yes
K236del	CMT1	11.09	5.2 ± 1.0	+++	7860.0	7862.17	-2.17	yes

*All proteins, including wt-P0ct, contain the C182L mutation.

**Values determined from protein sequences using ProtParam

***Expressed as mg protein obtained on average per liter culture. See S1 Fig for graphical representation.

**Fig 2:**
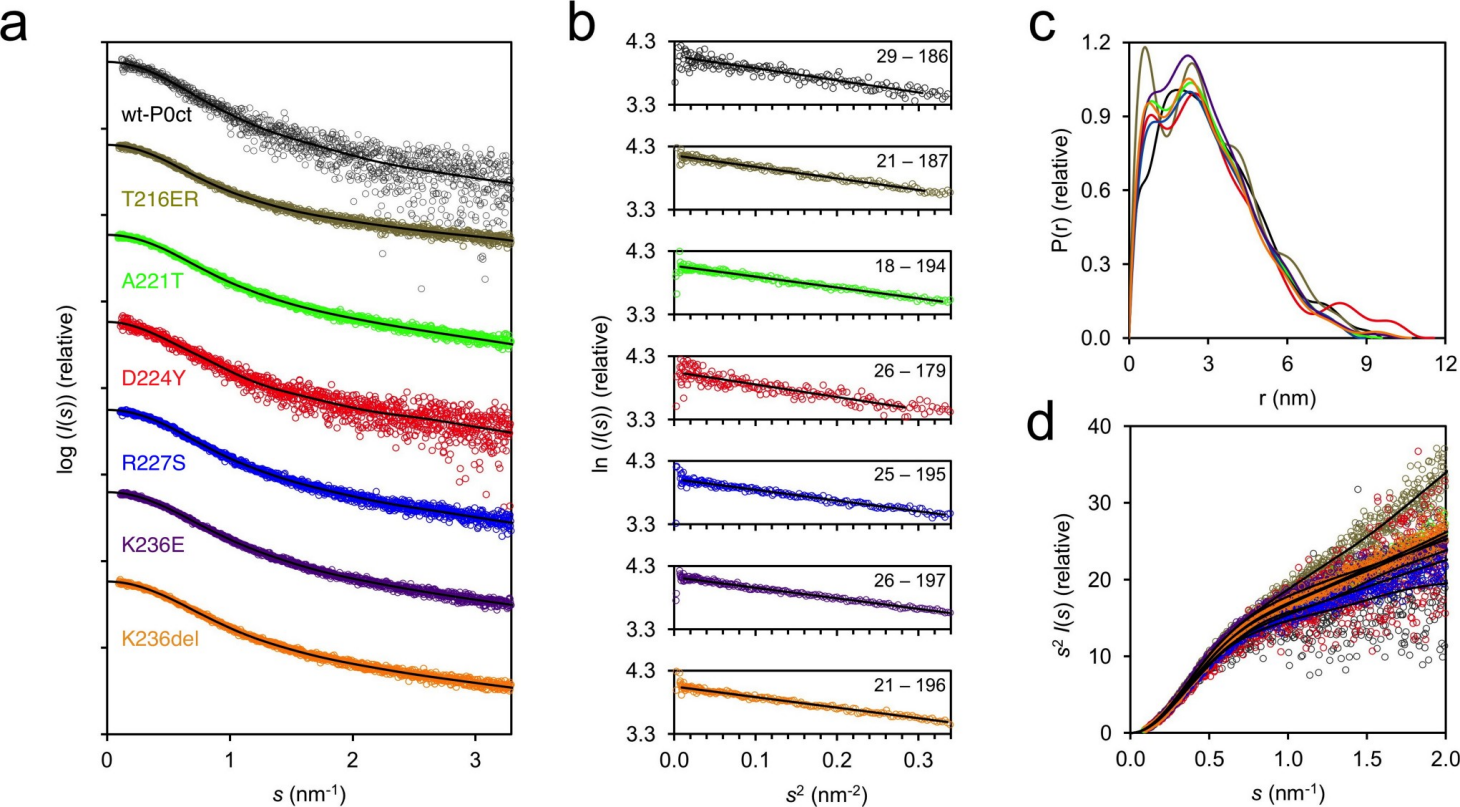
SAXS analysis of P0ct in solution. (a) SAXS data for P0ct variants. The scattering curves have been offset for clarity. (b) Guinier fits based on SAXS data. Data range is shown within each graph. (c) Distance distributions. (d) Kratky plots. P0ct variant data point coloring is consistent throughout the figure. GNOM fits to the data are shown as black lines in panels (a) and (c).

Small-angle X-ray scattering (SAXS) verified that for most variants, both the size and behaviour in solution were nearly identical, with radius of gyration (*R*_g_) and maximum dimension (*D*_max_) at 2.4–2.7 nm and 9.0–10.7 nm, respectively, and molecular masses matching monomeric protein based on *I*_0_ values (Fig 2, S2 Table). D224Y presented a marginally larger *D*_max_ (11.6 nm) compared to the other variants, but all variants were flexible and extended in solution, as evident from the Kratky plot (Fig 2D).

### The folding and lipid binding properties of P0ct CMT mutants

**Fig 3:**
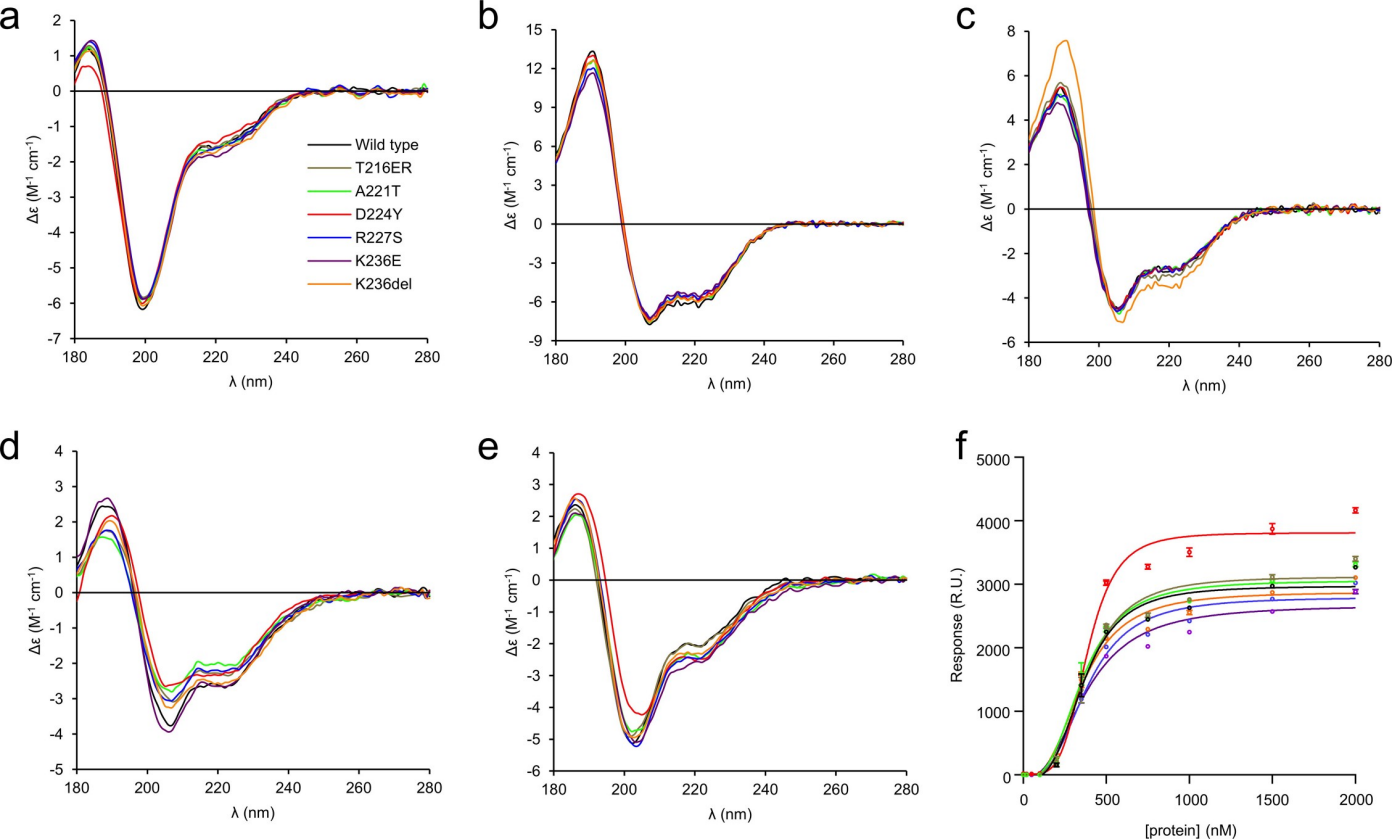
Folding and lipid binding analysis of P0ct variants. The folding of wt-P0ct and mutants was studied using SRCD spectropolarimetry in (a) water, (b) 30% TFE, (c) 0.5% SDS, (d) DMPC:DMPG (1:1), and (e) DMPC:DMPG (4:1) at 1:200 P/L ratio in each lipid condition. Additional spectra are presented in S2 Fig. (f) SPR measurements were used to determine the affinity of each P0ct variant to immobilized DMPC:DMPG (1:1) vesicles. The colour coding legend in panel (a) for each mutant trace also corresponds to all other traces in subsequent panels.

To compare the conformation of the P0ct variants, we carried out a series of synchrotron radiation circular dichroism (SRCD) spectroscopic experiments in the absence and presence of different lipid compositions, detergents, and 2,2,2-trifluoroethanol (TFE), as previously described for wt-P0ct [5]. P0ct is disordered in solution and gains a significant amount of secondary structure upon binding to small unilamellar vesicles (SUV) with a net negative surface charge [4,5]. In water, all mutants were disordered as expected, with D224Y having less secondary structure than the others (Fig 3). This is in agreement with the longer *D*_max_ determined using SAXS. All mutants closely resembled wt-P0ct in TFE and the detergents sodium dodecyl sulphate (SDS), *n*-dodecylphosphocholine (DPC), lauryldimethylamine *N*-oxide (LDAO), and *n*-octyl glucoside (OG) (Fig 3, S2 Fig). K236del was more α-helical than the other variants in the presence of SDS (Fig 3C), as evidenced by both the larger spectral amplitude and the slight change in peak position towards a higher wavelength.

Addition of DMPC retained the proteins in a disordered state, with D224Y deviating slightly (S2 Fig). In the presence of net negatively charged SUVs composed of DMPC:DMPG ratios of 1:1, 4:1, and 9:1, the variants presented some folding differences (Fig 3, S2 Fig). Overall, most folding was observed in 1:1 DMPC:DMPG, and the degree of folding decreased with decreasing fraction of DMPG, *i*.*e*. negative charge. In DMPC:DMPG (1:1), a small shift to the right of the maximum at 188 nm was evident for D224Y and K236del, indicating slightly increased folding, although the two minima at 208 and 222 nm, typical for helical content, remained the same for all variants (Fig 3D). In DMPC:DMPG (4:1), this effect was only observed for D224Y (Fig 3E). In DMPC:DMPG (9:1), the differences in signal magnitude were large, reflecting different levels of turbidity (S2 Fig). It can be assumed that the variants showing high turbidity under this condition are membrane-bound, while the ones giving strong CD signal of an unfolded protein do not bind to 9:1 DMPC:DMPG.

The affinity of P0ct variants towards immobilized DMPC:DMPG (1:1) SUVs was investigated using surface plasmon resonance (SPR). All variants bound to lipids with similar kinetic parameters (Fig 3F, Table 2), including the *A*_1_ value, which corresponds to the apparent *K*_d_, of 0.35–0.4 μM. This value in the same range with those obtained earlier for wt-P0ct, MBP, and P2 [5,20,22,23]. While the differences in *K*_d_ were minor, the behaviour of D224Y was unique: the observed maximal response level was higher compared to the other variants. This suggests that the D224Y variant can either accumulate onto immobilized vesicles in higher amounts, or it induces a change on the surface that affects the measurement, such as the fusion, swelling, or aggregation of lipid vesicles.

**Table 2:** SPR fitting parameters.

Variant	R_hi_	R_lo_	A_1_	A_2_	R^2^
wt-P0ct	2975 ± 79	-69.1 ± 61.5	363.2 ± 15.1	3.24 ± 0.41	0.986
T216ER	3123 ± 86	-44.6 ± 64.2	375.1 ± 15.6	3.17 ± 0.41	0.985
A221T	3061 ± 82	-44.8 ± 62.3	357.0 ± 15.5	2.97 ± 0.36	0.986
D224Y	3811 ± 81	11.3 ± 66.3	385.2 ± 11.3	4.42 ± 0.54	0.989
R227S	2798 ± 78	-39.5 ± 55.2	384.9 ± 16.0	2.94 ± 0.36	0.986
K236E	2671 ± 92	-49.5 ± 60.0	380.8 ± 20.0	2.53 ± 0.34	0.983
K236del	2880 ± 79	-33.9 ± 58.9	356.1 ± 16.0	2.85 ± 0.35	0.986

### Effect of CMT mutations on lipid membrane properties

**Fig 4:**
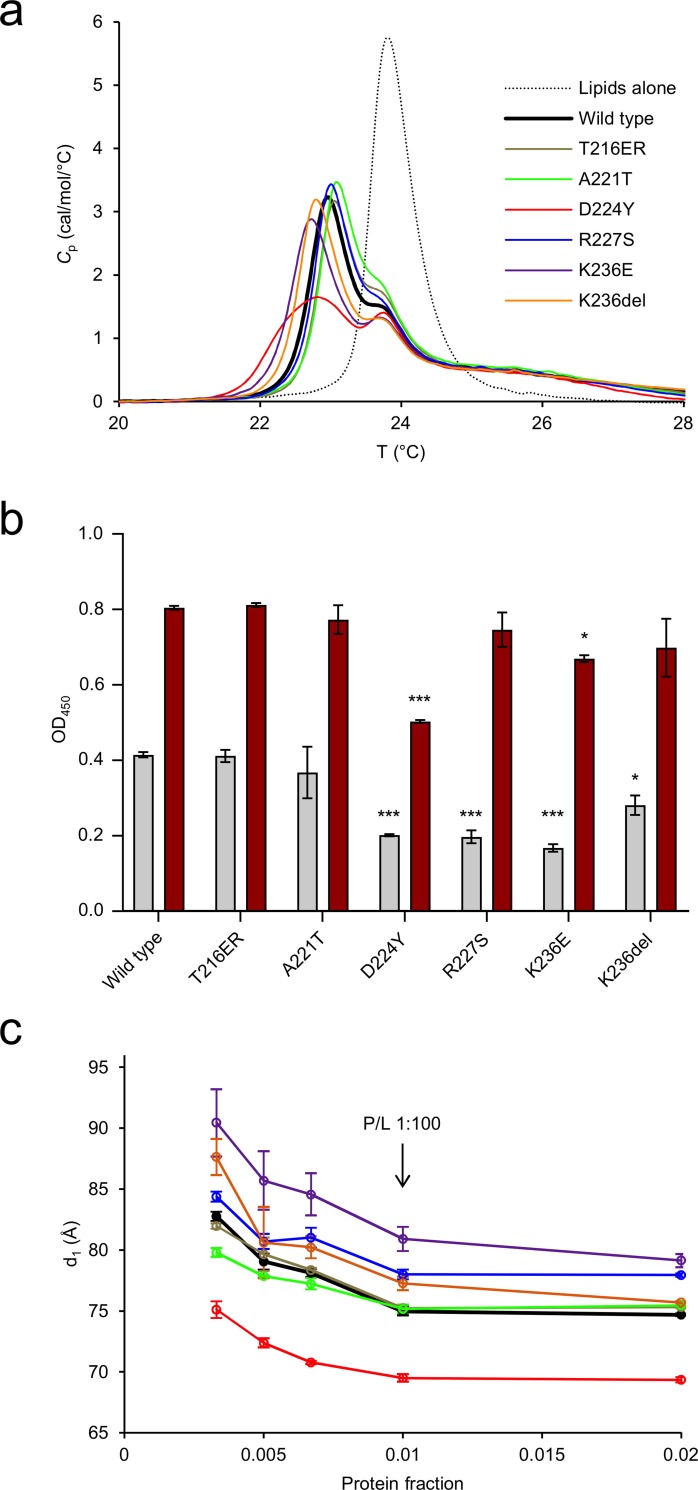
Analysis of protein-induced lipid structure behaviour. (a) DSC analysis of lipid phase transition. The experiments were carried out at 350 μM DMPC:DMPG (1:1) and a 1:100 P/L ratio. (b) Turbidimetric analysis of 0.5 mM DMPC:DMPG (1:1) at 5 μM (gray) and 10 μM protein concentration (dark red). These proteins concentrations translate to 1:100 and 1:50 P/L ratios, respectively. Error bars represent standard deviation. Statistical analysis was performed using one-way analysis of variance (ANOVA) followed by Dunnett’s multiple comparisons test to wt-P0ct turbidity within the same protein concentration series (*: P < 0.05; ***: P < 0.001). (c) SAXD analysis reveals that D224Y displays a significantly tighter mean repeat distance compared to wt-P0ct, whereas K236E is most loose. The traces have identical colouring to (a).

To determine the effect of the mutations on lipid structure, experiments probing changes in the thermodynamic and structural properties of lipid membranes were carried out. As shown before [5], the presence of P0ct changes the melting behaviour of dimyristoyl lipid tails, inducing a population that melts 0.9°C below the major phase transition temperature of 23.8°C. The presence of the mutations altered this effect mildly (Fig 4A), with T216ER and R227S behaving similarly to wt-P0ct. The Lys236 mutations deviated from wt-P0ct, with a decreased temperature for the emerged population; K236E and K236del showed lipid phase transition temperatures of ~22.8°C. A221T presented a slightly higher temperature for phase transition compared to wt-P0ct, with the major peak at 23.1°C. Based on the shape of the calorimetric landscape, D224Y was clearly different from the rest, as the new population did not appear as a single, sharp, symmetric peak, but was rather formed of several overlapping peaks. The fitted phase transition temperatures and associated errors are given in S3 Table.

Similarly to MBP and P2 [20,22], P0ct is capable of inducing concentration-dependent solution turbidification, when mixed with lipid vesicles of net negative charge [5]. The turbidity can arise from vesicle fusion and/or aggregation, and different processes may be dominant in different samples with respect to the measured signal. To determine the effect of P0ct CMT mutations on this function, turbidity experiments were carried out with the different variants. T216ER and A221T produced turbidity levels similar to wt-P0ct (Fig 4B, S3A Fig). At 1:100 P/L ratio, D224Y, R227S, K236E, and K236del all had decreased turbidity. At a P/L ratio of 1:50, however, only D224Y had a significant inhibitory effect on turbidity. This result highlights that the D224Y mutant protein may function differently from the other variants, when it binds to and aggregates vesicles.

To shed further light on the protein-induced changes in membrane structure, small-angle X-ray diffraction (SAXD) experiments were performed on P0ct-membrane mixtures. In our earlier study, wt-P0ct mixed with lipids produced two strong Bragg peaks, and the corresponding repeat distance evolved as a function of the P/L ratio [5]. Here, we observed that in all cases, the repeat distance increased when protein concentration in the sample decreased (Fig 4C, S3B Fig). Each variant presented a minimum repeat distance, which was reached at and above a P/L ratio of 1:100. The repeat distance for wt-P0ct was ~7.5 nm, while D224Y produced a spacing of <7.0 nm. R224S, K236E, and K236del had looser packing than wt-P0ct. K236E had a minimum repeat distance of ~8.0 nm at the highest protein concentration.

**Fig 5:**
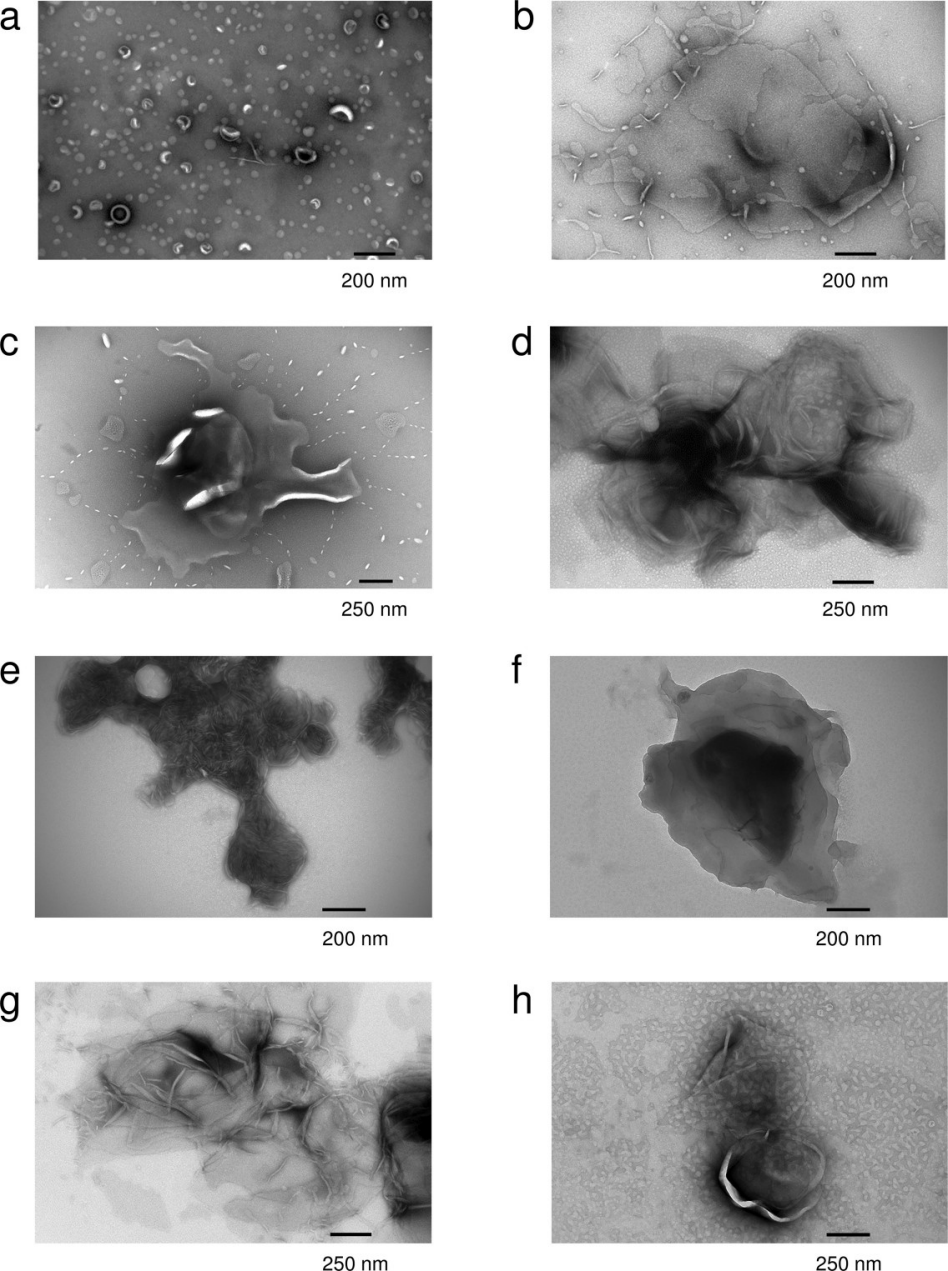
EM analysis of P0ct mutants. Negatively stained samples of DMPC:DMPG (1:1) vesicles were imaged (a) alone, and with (b) wt-P0ct, (c) T216ER, (d) A221T, (e) D224Y, (f) R227S, (g) K236E, and (h) K236del at a 1:200 P/L ratio. D224Y forms multilayered lipid structures that are absent for wt-P0ct.

To understand the effect of the mutations on the function of P0ct, and the origin of the high molecular order reflected by X-ray diffraction, electron microscopy imaging was performed. Most mutants functioned in a manner similar to wt-P0ct, producing large vesicular structures with a spread-out morphology (Fig 5), with occasional regions indicative of bilayer stacking. D224Y showed a clear difference to wt-P0ct, producing strongly stacked myelin-like membranes in a manner resembling MBP [20]. This gain of function was reproducible over a wide range of P/L ratios (S4 Fig) and a unique feature among the six mutant P0ct variants. The results confirm that the Bragg peaks seen in SAXD, indeed, originate from repeat distances in membrane multilayers, identically to two other PNS myelin peripheral membrane proteins, MBP and P2 [20,22,24]. The observed bilayer spacing for the D224Y mutant in EM was narrow and in general better defined than seen for MBP [20], suggesting that P0ct forms a tight structure within and/or between the membranes. Based on SAXD, the intermembrane spacing is ~3 nm, a value in close relation to the dimensions of the major dense line (MDL) in myelin *in vivo*.

**Fig 6:**
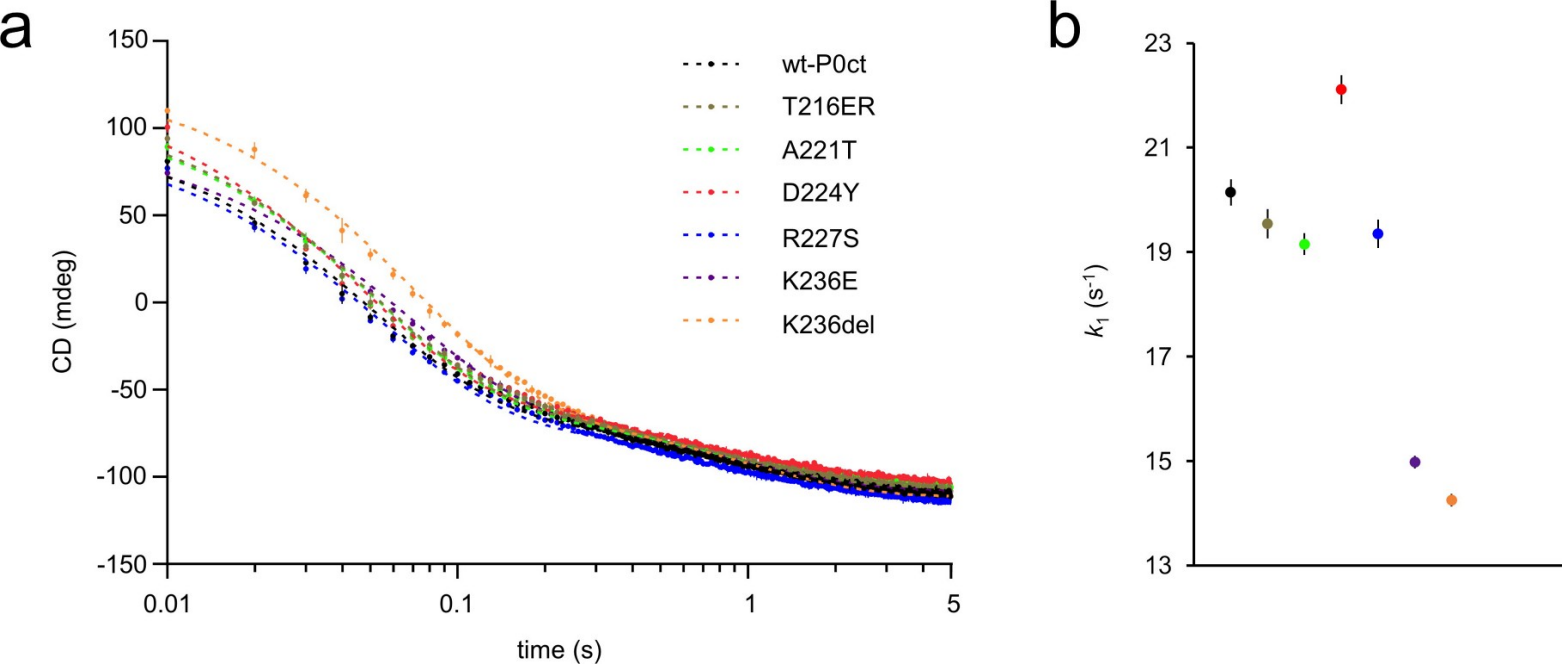
SRCD stopped-flow kinetics of protein-induced initial lipid turbidification. (a) The SRCD signal at 195 nm was monitored using rapid kinetics for 5 s. wt-P0ct and mutants were mixed with DMPC:DMPG (1:1) lipids at 1:200 P/L ratio in the presence of 150 mM NaF. Fits (dashed lines) are plotted over the measurement points. Error bars represent standard deviation. See Table 4 for fitting results. (b) Graphical comparison of the obtained *k*_1_ values.

To gain insight into the kinetic aspects of P0ct-induced lipid fusion/aggregation, stopped-flow kinetics experiments were performed using SRCD (Fig 6, Table 3) [21]. All variants followed a similar kinetic pattern as wt-P0ct and could be best fitted to a two-phase exponential decay with two rate constants (*k*_1_, fast and *k*_2_, slow). Rather minor differences were present: *k*_2_ values were very similar in all cases, and while D224Y presented 10% higher *k*_1_ and *k*_1_/*k*_2_ compared to wt-P0ct and most other variants, both K236E and K236del displayed *k*_1_ and *k*_1_/*k*_2_ 20% lower than for wt-P0ct, indicating slower kinetics (Fig 6B). While all variants produced a similar end-level CD value around -100 mdeg at 195 nm wavelength, the starting level for K236del was higher than for any other variant, and remained so until ~0.3 s, before settling on a level similar to other variants. It is currently unclear whether this is due to an increased level of protein folding or less scattered light from fused or aggregated vesicles.

**Table 3:** Kinetic constants for protein-induced vesicle turbidity. The kinetic constants were obtained by fitting the data to a two-phase exponential decay function. All errors represent standard deviation.

Variant	*k*_1_ (s^-1^)	*k*_2_ (s^-1^)	*k*_*1*_*/k*_*2*_	*R*^2^
wt-P0ct	20.14 ± 0.25	1.12 ± 0.01	17.96 ± 0.22	0.993
T216ER	19.54 ± 0.28	1.18 ± 0.02	16.63 ± 0.25	0.991
A221T	19.15 ± 0.21	1.14 ± 0.01	16.76 ± 0.20	0.994
D224Y	22.11 ± 0.28	1.19 ± 0.02	18.53 ± 0.25	0.992
R227S	19.35 ± 0.27	1.08 ± 0.02	17.95 ± 0.26	0.992
K236E	14.98 ± 0.12	1.02 ± 0.01	14.64 ± 0.13	0.997
K236del	14.25 ± 0.12	1.05 ± 0.01	13.54 ± 0.13	0.997

### The membrane insertion mode of P0ct

**Fig 7:**
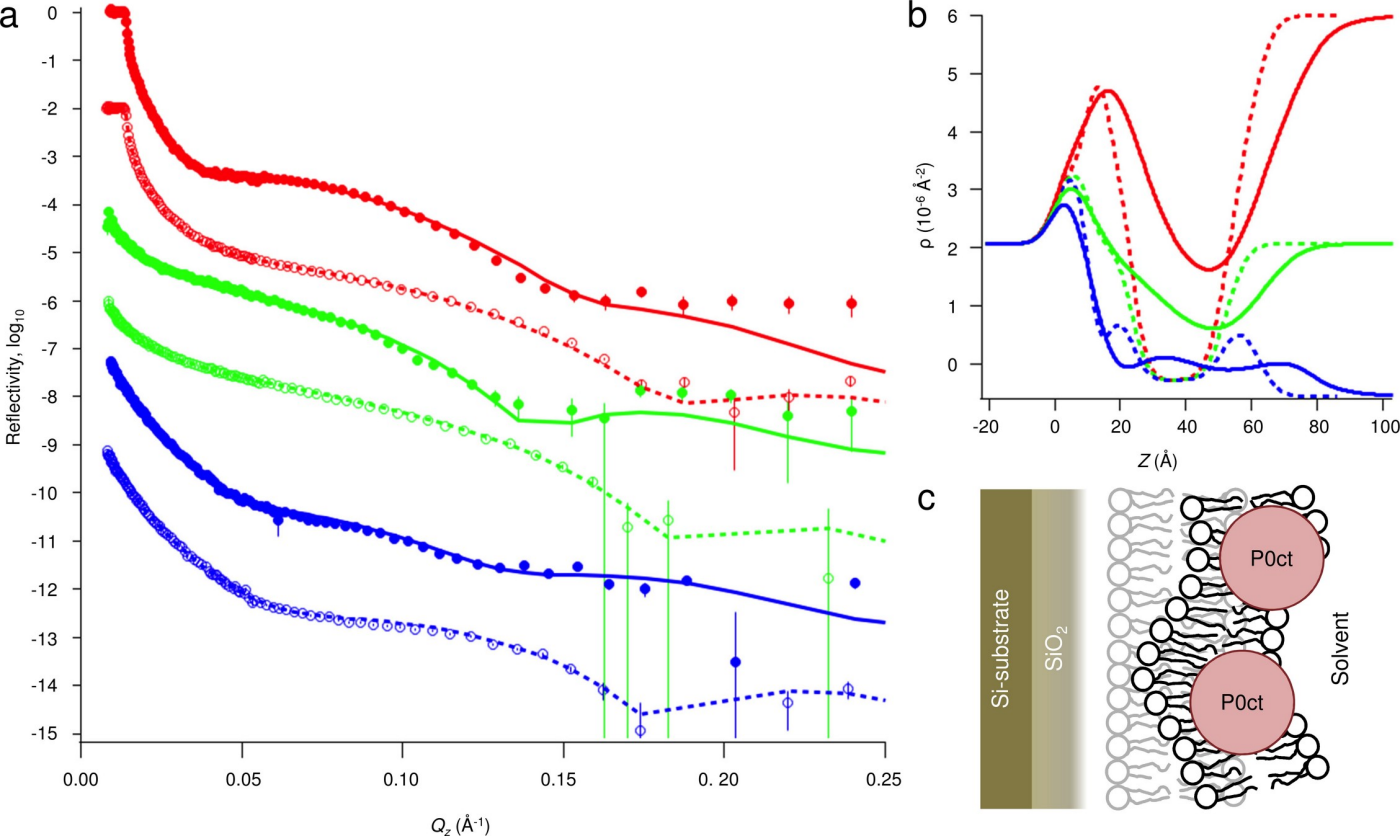
Neutron reflectometry. (a) NR data for a supported DMPC:DMPG (1:1) bilayer before (open markers) and after incubation with wt-P0ct (closed markers). The solvent contrasts used were 95% D_2_O (red), Si-matched water (SMW, 38% D_2_O; green), and 100% H_2_O (blue). The error bars denote standard deviation. Fits are shown as dashed and solid lines for the bilayer before and after addition of wt-P0ct, respectively. (b) Scattering length density (ρ) profiles obtained from the fitting. (c) Model for the P0ct-bound membrane. The protein-free membrane is shown in light gray on the background.

To understand the membrane insertion of P0ct, how it compares to MBP [20], and how it might be related to disease mutations, we performed neutron reflectometry (NR) experiments (Fig 7, Table 4). The insertion of P0ct to a DMPC:DMPG SLB was quite different to that of MBP. P0ct inserted completely into the membrane, thickening it by 2 nm and increasing its roughness, most likely due to increased bilayer mobility, as the hydration layer below the membrane became thicker (Fig 7B and 7C). P0ct was present in the acyl tail fraction of the membrane, as well as in the outer headgroup fraction. The data could not be fitted with only these parameters, but a very rough, narrow layer of protein had to be considered on top of the membrane. Unfortunately, the roughness and high solvation fraction of this layer did not allow for precise thickness determination: the layer was modelled to be between 5–15 Å thick within the fit to the data. To investigate the effect of the D224Y mutation on P0ct membrane association, NR data were collected for SLB-bound D224Y, which appeared identical to wt-P0ct (S5 Fig).

**Table 4:** NR fitting parameters. The fits and obtained scattering length density profiles are shown in Fig 7.

		Bilayer alone	Bilayer with 0.5 μM wt-P0ct
**Substrate**	Oxide thickness (Å)	10.6	11
	Oxide solvation (%)	0	0
	Oxide roughness (Å)	4	4
	Hydration layer between oxide and bilayer (Å)	4.6	12
	Hydration layer roughness (Å)	3	6
**Bilayer**	Bilayer area-per-molecule (Å^2^/molecule)	60	70
	Inner headgroups thickness (Å)	8.3	8
	Inner headgroups roughness (Å)	3.6	8.1
	Inner headgroups solvation (%)	35	45
	Acyl tails thickness (Å)	28.8	32
	Acyl tails roughness (Å)	3.8	13.3
	Acyl tails hydration (%)	0	17
	Outer headgroups thickness (Å)	8.8	8
	Outer headgroups roughness (Å)	4.9	9.5
	Outer headgroups solvation (%)	35	53.5
**wt-P0ct**	Protein in inner headgroups (%)	-	0
	Protein in acyl tails (%)	-	10
	Protein in outer headgroups (%)	-	20
	Protein layer thickness (Å)	-	7
	Protein layer roughness (Å)	-	15
	Protein layer solvation (%)	-	86

### Sequence analysis and modelling

**Fig 8:**
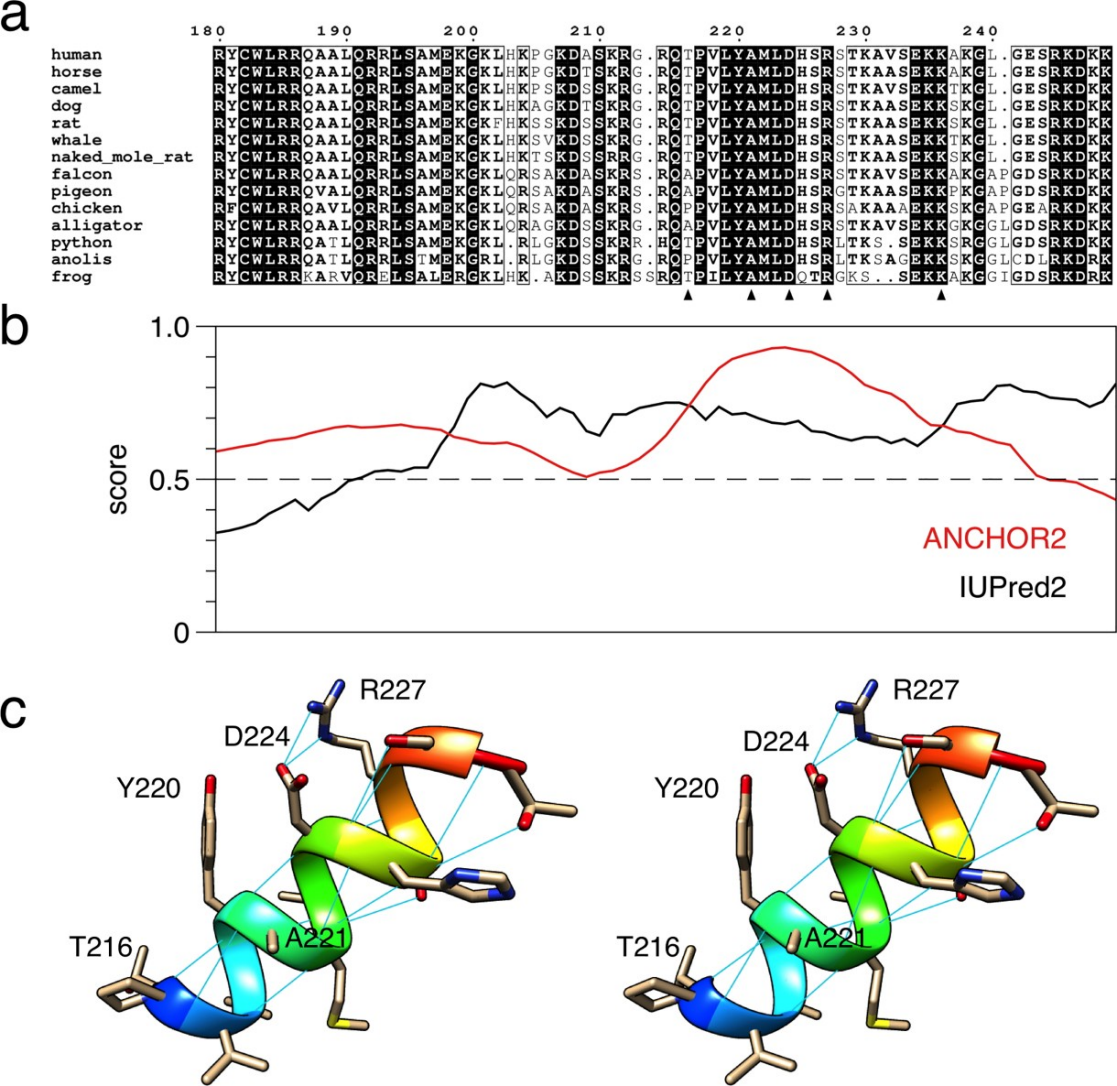
Sequence analysis of P0ct. (a) Alignment of P0ct from selected vertebrates. The locations of the P0ct mutations are indicated with arrowheads. (b) ANCHOR2/IUPred2 analysis of P0ct highlights a region likely to fold upon ligand binding between residues 215–230. (c) Stereo view of a model of the predicted membrane-binding helical region. Hydrogen bonds are shown with thin blue lines.

For a further insight into the molecular basis of the differences between P0ct variants, we carried out computational analyses on P0ct sequence and folding. A sequence alignment of P0ct from different vertebrate species (Fig 8A) showed full conservation of certain segments of the sequence; specifically, all mutated residues studied here with the exception of Thr216 are fully conserved across the species studied. The region carrying the mutations, corresponding to the neuritogenic segment and predicted to fold into a membrane-binding helix, is strongly predicted to fold upon binding (Fig 8B). Our earlier data showed that this peptide segment inserts into the membrane in a tilted helical conformation [5]. In the predicted membrane-embedded tilted helix, Asp224 and Arg227 are on the same face of the helix, next to Tyr220 (Fig 8C). In such a helix, Asp224 and Arg227 are likely to form a salt bridge, while the other end of the helix would be embedded in the membrane.

**Fig 9:**
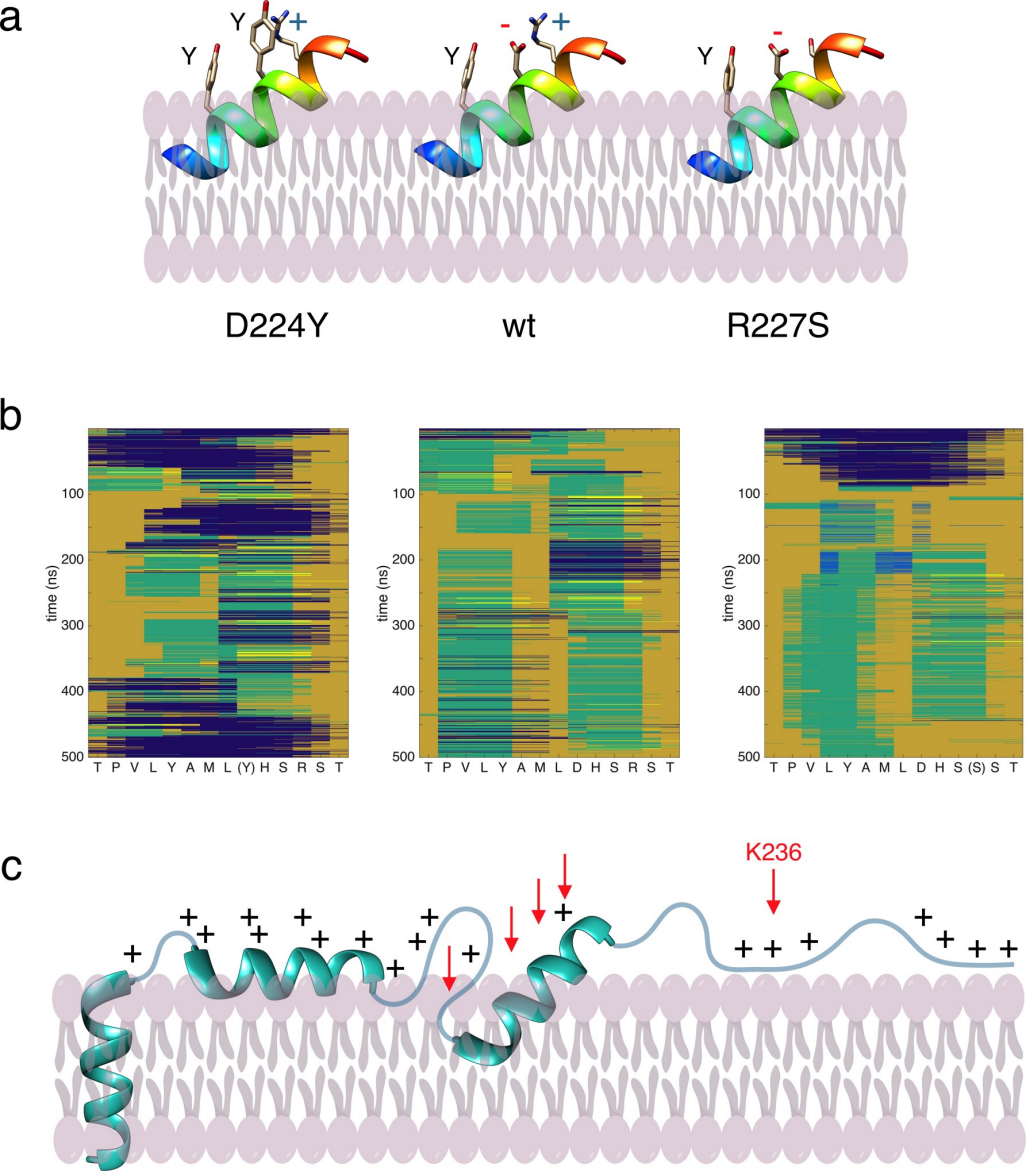
Models for P0ct. (a) Tentative models of the membrane-embedded P0ct helix with the two mutations having opposite effects on membrane stacking. The tilted orientation is estimated based on earlier oriented CD experiments on wt-P0ct [5]. (b) MD simulations starting from a helical structure in water. Blue, α helix; orange, coil; green, turn; light blue, β strand; yellow, 3_10_ helix. (c) A working model for the binding of P0ct to the membrane cytosolic surface, based on current knowledge and predictions. It is likely that the C-terminal region, including Lys236, is less tightly bound to the surface. Positions of the mutations studied in the current work are indicated with red arrows.

The effects of the mutations D224Y and R227S can be partly rationalized based on this simple model (Fig 9A). D224Y would result in two Tyr residues next to each other facing the apposing membrane together with an Arg residue—this could change the membrane properties, such that stacking interactions are stronger and hypermyelination can occur. On the other hand, R227S will expose a free negative charge on Asp224, which should be repulsive towards an apposing negatively charged bilayer surface. When the region 216–229 is modelled as a helix and subjected to MD simulations in water, differences in folding propensity are observed with these mutations (Fig 9B). While wild-type and R227S rapidly lose helical conformation, D224Y remains mainly helical throughout the 500-ns simulation, possibly indicating a more stable helix in the mutant protein. Whether this is the case for a membrane-embedded helix in P0ct, is currently unknown. A schematic model of P0ct on a membrane surface is shown in Fig 9C.

## Discussion

The formation of compact myelin and the major dense line requires an interplay of myelin proteins, many of which have similar functional properties despite lack of sequence homology. Considering the MDL of PNS compact myelin, the major protein components according to current knowledge are MBP, P2, P0ct, and cytosolic loops of PMP-22. We characterized the potential functional anomalies of P0ct CMT mutants in membrane binding using earlier established biophysical strategies [5,20,21].

The six mutations reported in P0ct are clustered within or near the neuritogenic segment. Most of them reside in the vicinity of putatively phosphorylated Ser residues (Fig 1A), which may be affected by P0ct mutations [10,25]. Many P0 mutations have been suggested to lead to UPR activation [17–19], indicating problems with translation rate, folding, and/or membrane insertion. Given the fact that P0ct is known to interact with lipid membrane surfaces [4,5,21], mutations within P0ct could also have direct effects on the formation of mature compact myelin at the molecular level.

### Mechanism of P0ct binding to membranes

In order to fully understand the effects of P0ct mutations on its structure and function, detailed knowledge about P0ct binding to lipid membranes, and the effects thereof on multilayered membrane stacks, is required. NR allowed us to gain a picture of P0ct in a lipid bilayer. P0ct inserts deep into a membrane, with only a small fraction remaining solvent-exposed on the membrane surface. This is a clear difference to MBP, which forms a brush-like protein phase on top of the membrane surface, while being partially embedded into the bilayer [20]. After undergoing charge neutralization and folding, P0ct seems to collapse into a tight conformation and remain stable. The compact, deep conformation of P0ct suggests that instead of directly embedding into two bilayers, which is the working model for *e*.*g*. MBP-induced stacking [20,26], P0ct may change the surface properties of the membrane in a way that supports apposing bilayer surface adhesion. It could also regulate membrane curvature and the twining of lipid bilayers around the axon.

At the level of full-length P0, P0ct is a direct extension of the transmembrane segment, and hence, anchored permanently to a membrane surface at its beginning. Membrane stacking could, in theory, involve the insertion of P0ct across the MDL into an apposing membrane leaflet, which is only 3 nm away. Considering this scenario, it must be taken into account that P0 is basally expressed in Schwann cells even before myelination occurs [27]. Moreover, P0 is translated and inserted into the ER membrane and trafficked through the trans-Golgi network to the plasma membrane after the Ig-like domain has been post-translationally modified [28,29]. If P0ct were to enter an apposing membrane during the formation of compact myelin, it would have to remain in a disordered state until another membrane is present. On the other hand, if P0ct is embedded in the membrane after translation, it might afterwards be able to dissociate and enter the apposing leaflet within compact myelin. Considering the attractive phospholipid bilayer around the transmembrane helix, and the fact that P0ct binds negatively charged lipids essentially irreversibly *in vitro* [5], both mechanisms described above are unlikely to exist. Thus, the role of P0ct in membrane adhesion is likely to be based on altered lipid membrane properties, as opposed to MBP and P2, which directly interact with two membrane surfaces. While P2 and MBP synergistically stack lipid bilayers *in vitro* [30], mice lacking both proteins formed apparently normal and functional myelin [31]. Hence, multiple factors must participate in the correct formation of compact myelin; these include both the lipid components of the myelin membrane, different myelin proteins, as well as signalling molecules and inorganic ions. Hence, further experiments in more complex sample environments are required to decipher the details of the molecular interplay between these factors in PNS myelin MDL formation.

### P0ct mutations and membrane interactions

Compared to wt-P0ct, we observed only subtle differences for two mutants: T216ER and A221T. While T216ER behaved very similarly to wt-P0ct, its role in CMT etiology could be of another origin than related to protein-membrane binding. A221T, on the other hand, resides in the YAML-motif, which directs the trafficking of P0 [32] and might compromise the function of P0 even without inducing changes in membrane binding, especially when combined with a second mutation in the extracellular domain, such as the deletion of Val42 [16].

Functionally, the most interesting mutant studied here is D224Y, which has been described in at least 3 studies [11–13]. Our results show it is a gain-of-function mutant, inducing ordered lipid bilayer stacks *in vitro*, which are more tightly packed than those formed by wt-P0ct or the other variants. Importantly, the affinity of the tightly packed membrane bilayers with the D224Y mutant towards each other is high enough to allow visualization of the ordered multilayers by EM, which was a striking difference to all other variants studied. The results correlate well the hypermyelinating disease phenotype [11]. Neutron reflectometry produced a nearly identical result for D224Y compared to wt-P0ct, which together with the SRCD experiments indicates that the conformation and level of insertion of wt-P0ct and D224Y are similar in the membrane. Small changes, not detectable by these methods, may however occur. The change of an acidic to an aromatic residue near the lipid bilayer surface could enable a specific interaction between surfaces that results in the observed gain of function. In this case, two vicinal aromatic residues on P0ct (Fig 9A) might enhance membrane interactions, perhaps in a fashion similar to the tandem Phe motifs in MBP [33]. The fine molecular details of the effects causing tighter membrane stacking by the D224Y mutant protein, however, remain uncovered at the moment. There could exist differences in the orientation of the embedded helix in wt-P0ct and D224Y, for example. Both electrostatic forces, hydrophobic interactions, and aromatic stacking are likely involved in the altered proteolipid multilayer. Interactions of the protein with both of the apposing membranes could be affected by the mutation directly or indirectly, and these effects may be linked to the observed differences in the physicochemical behaviour of the lipid bilayer itself.

P0 is the most abundant protein in PNS myelin [34,35], contributing primarily to the formation of the intraperiod line [36], and molecular mechanisms of D224Y-induced tight stacking could be two-fold. Firstly, with its short repeat distance– 1–2 nm smaller compared to MBP and P2 based on SAXD [5,22,24]–and active membrane binding, as evident from SPR, the mutant might cause size exclusion of P2 and other factors out of the cytoplasmic stack, leading to defective compact myelin maintenance. In PNS compact myelin, P2 is even more abundant in the cytoplasmic compartment than MBP, can form membrane stacks, and harbours a maintenance role in myelin homeostasis as a lipid carrier [22,31]. Secondly, the tendency of D224Y to form such ordered, tight systems might affect the Ig-like domains on the extracellular side. In the hypermyelinating phenotype of D224Y patients, membrane stacking seems condensed and regular, without abnormally loosened myelin [11]. SPR indicates that more D224Y can accumulate on membranes, and full-length P0 D224Y could accumulate and tighten up within the membrane, causing the intraperiod line to become more crowded and/or structured. The original discovery of the D224Y mutation [11] suggested that it has a gene dosage effect, since heterozygous carriers presented little to no symptoms. Hence, the presence of wild-type P0 can rescue the effects of the mutation. Correct gene dosage of P0 is important for normal myelination in animal models as well as CMT patients [11,37–41]. The molecular details of the involved mechanisms are currently lacking. Further studies on the D224Y mutation both *in vitro*, *in silico*, and *in vivo* will help in understanding molecular aspects of both normal and abnormal myelination.

Lys236 appears to be a functionally important amino acid in P0ct. In its membrane-bound state, P0ct is likely to have Lys236 close to the lipid headgroups (Fig 9C), and altering the charge in this environment might influence folding and the global positioning of P0ct on the membrane. Indeed, a gradual effect in membrane packing was observed in SAXD; the repeat structure loosens, as residue 236 neutralizes (K236del) and turns to negative (K236E). Turbidimetry also indicated a clear effect of charge reversal at residue 236. The Lys236 mutants folded to a similar degree as wt-P0ct, which suggests that the role of Lys236 is in membrane packing, rather than protein folding. This is supported by the slower kinetic parameters for Lys236 mutants in stopped-flow SRCD measurements.

Similarly to Lys236, Arg227 could harbour a role in membrane packing. In our experiments, R227S is one of the mutants that appeared to induce weaker adhesion than the wild-type protein. The mutation results in a loosened repeat structure without a major impact on protein folding, which could relate to the exposed negative charge of Asp224 causing repulsion towards an apposing bilayer. Arg227 might be involved in electrostatic anchoring of the protein to the lipid headgroups–the R227S mutation likely has low impact on ER stress and UPR, as mutated P0 correctly localizes to the plasma membrane [42].

### Concluding remarks

To a large extent, the P0ct CMT variants studied here perform similarly to wt-P0ct in controlled simple environments. This might differ *in vivo*, where other components are present and P0 is present in its full-length form. Our characterization is focused on protein-lipid interactions and does not take into account possible protein-protein interactions with MBP, P2, or PMP22, which might be relevant for myelination and disease phenotypes. Nevertheless, we have uncovered critical amino acids in P0 that may contribute to the formation of healthy myelin and be involved in disease mechanisms. These include Arg227, Lys236, and Asp224. Our results shed light on the molecular fundamentals of myelination in the PNS, but more comprehensive studies in biological model systems, as well as on molecular structure and dynamics of native-like myelin membranes, are needed for deciphering the mechanisms of the P0ct mutations causing human neuropathy.

## Experimental procedures

### Bioinformatics, mutagenesis, protein expression & purification

Secondary structure prediction for P0ct was performed using JPred [43]. Binding-induced folding was predicted with ANCHOR2/IUPred2 [44]. Mutations were generated in the P0ct pHMGWA expression vector [5,45] by PCR using Phusion High-Fidelity DNA polymerase (Thermo Fisher Scientific) with 5′-phosphorylated primers that introduced the desired point mutations. The samples were treated with *Dpn*I (New England Biolabs) to digest template DNA and linear vectors circularized using T4 DNA ligase (New England Biolabs), followed by transformation and plasmid isolation. The presence of mutations and integrity of the constructs was verified using DNA sequencing.

Protein expression and purification were carried out in *E*. *coli* BL21(DE3) as described for wt-P0ct [5], with the exception of an added amylose-resin affinity step between Ni-NTA and size-exclusion chromatography. The step was introduced to remove any contaminating maltose-binding protein tags from the tobacco etch virus protease-digested recombinant proteins. Size exclusion chromatography was carried out using Superdex S75 16/60 HiLoad and Superdex 75 10/300GL columns (GE Healthcare) with 20 mM HEPES, 150 mM NaCl, pH 7.5 (HBS) as mobile phase, with the exception of D224Y, where a 20 mM Tris-HCl, 300 mM NaCl, pH 8.5 (TBS) solution was used. The monodispersity and *R*_h_ of all proteins were checked from filtered 1 mg/ml samples using a Malvern Zetasizer ZS DLS instrument. The D224Y mutant was then dialyzed into HBS. Additionally, all proteins were dialyzed into water prior to SRCD experiments.

### Mass spectrometry

The molecular weight and identity of the purified proteins were verified by mass spectrometry. In short, the proteins were subjected to ultra-performance liquid chromatography (UPLC) coupled electrospray ionization (ESI) time-of-flight mass spectrometry in positive ion mode, using a Waters Acquity UPLC-coupled Synapt G2 mass analyzer with a Z-Spray ESI source. This allowed us to determine the undigested masses of each purified P0ct variant. Protein identity and the presence of the desired mutations were confirmed from peptides extracted after in-gel tryptic proteolysis, using a Bruker Ultra fleXtreme matrix-assisted laser desorption/ionization time-of-flight (MALDI-TOF) mass analyzer.

### Small-angle X-ray scattering

SAXS data were collected from protein samples at 0.3–12.9 mg/ml in HBS and TBS on the EMBL P12 beamline, PETRA III (Hamburg, Germany) [46]. Monomeric bovine serum albumin (M_r_ = 66.7 kDa; *I*_0_ = 499.0) was used as a molecular weight standard. Data were processed and analyzed using the ATSAS package [47], and GNOM was used to calculate distance distribution functions [48]. See S2 Table for further details.

### Vesicle preparation

DMPC, DMPG, and DOPC were purchased from Larodan Fine Chemicals AB (Malmö, Sweden). DOPS and the deuterated d_54_-DMPC and d_54_-DMPG were purchased from Avanti Polar Lipids (Alabaster, Alabama, USA).

Lipid stocks were prepared by dissolving dry lipids in chloroform or chloroform:methanol (9:1 v/v) at 10–30 mM. Mixtures were prepared from stocks at the desired molar ratios, followed by solvent evaporation under a stream of nitrogen and lyophilizing overnight at -52°C. The dried lipids were stored at -20°C or used directly for liposome preparation.

Liposomes were prepared by mixing dried lipids with water or HBS at 10–15 mM, followed by inverting at ambient temperature for at least 3 h. Multilamellar vesicles (MLV) were prepared by freeze-thaw cycles in liquid N_2_ and a warm water bath and vortexing. The cycle was performed 7 times in total. Large unilamellar vesicles (LUV) were prepared by passing fresh MLVs through a 0.1-μm membrane 11 times at 40°C. SUVs were prepared by ultrasonication of fresh MLVs using a probe tip sonicator (Sonics & Materials Inc. Vibra-Cell VC-130) until clarified. All lipid preparations were immediately used in experiments.

### Synchrotron radiation circular dichroism spectroscopy

SRCD spectra were collected from 0.1–0.5 mg/ml protein samples in water on the AU-CD beamline at ASTRID2 synchrotron (ISA, Aarhus, Denmark). Samples containing lipids were prepared right before measurement by mixing proteins (P/L ratio 1:200) with SUVs. 100-μm pathlength closed circular cells (Suprasil, Hellma Analytics) were used for the measurements. Spectra were recorded from 170 to 280 nm at 30°C. Baselines were subtracted and CD units converted to Δε (M^-1^ cm^-1^) in CDtoolX [49]. SDS and TFE were from Sigma-Aldrich and the detergents LDAO, OG, DM, and DPC from Affymetrix.

Rapid kinetic SRCD data were collected as described [21]. In short, an SX-20 stopped-flow instrument (Applied Photophysics) mounted on the AU-rSRCD branch line of the AU-AMO beamline at ASTRID2 (ISA, Aarhus, Denmark) at was used for data collection at 10°C. 1-to-1 mixing of a 0.1 mg/ml protein solution and a DMPC:DMPG (1:1) SUV solution (at P/L ratios 1:200) was achieved using a mixer (2 ms dead time) before injection into the measurement cell (160 μl total volume, 2-mm pathlength) per shot. The CD signal (mdeg) was monitored at a fixed wavelength of 195 nm for 5 s with a total of 5–10 repeat shots per sample, which were averaged into a single curve. Each sample was prepared and measured in duplicate. Water baselines were subtracted from sample data. The data were fitted to different exponential functions using GraphPad Prism 7.

### Surface plasmon resonance

SPR was performed on a Biacore T200 system (GE Healthcare). According to the manufacturer’s instructions, 100-nm LUVs of 1 mM DMPC:DMPG (1:1) were immobilized on an L1 sensor chip (GE Healthcare) in HBS, followed by the injection of protein solutions. Chip regeneration was performed using a 2:3 (v:v) mixture of 2-propanol and 50 mM NaOH. The protein concentration was 20–2000 nM in HBS, and a single concentration per lipid capture was studied; all samples were prepared and measured in duplicate. In each run, one sample was measured twice to rule out instrumental deviation. The binding response as a function of protein concentration was plotted and fitted to the 4-parameter model

$$R=R_{hi}-\frac{R_{hi}-R_{lo}}{1+{\left(\frac{\left[P0ct\right]}{A_{1}}\right)}^{A_{2}}}$$

to gain information about association affinity.

### Differential scanning calorimetry

Proteins were mixed with MLVs in HBS at a protein-to-lipid ratio of 1:100 or 1:250, always containing 350 μM of DMPC:DMPG (1:1) in a final volume of 700 μl. Lipid samples without proteins were prepared as controls. The samples were degassed for 10 min under vacuum with stirring at 10°C before measurements.

DSC was performed using a MicroCal VP-DSC calorimeter with a cell volume of 527.4 μl. The reference cell was filled with HBS. Each sample was scanned from 10 to 40°C and back to 10°C in 1°C/min increments. Baselines were subtracted from sample curves and zeroed between 15 and 20°C to enable straightforward comparison between samples. All samples were prepared and measured twice, with the observed trends being reproducible.

### Vesicle turbidimetry and X-ray diffraction

For turbidimetric measurements, SUVs of DMPC:DMPG (1:1) were mixed with 0.5–10 μM protein in duplicate and mixed thoroughly. Turbidity was recorded at 450 nm at 30°C using a Tecan Spark 20M plate reader. Turbidity of protein-free SUVs was subtracted from the protein samples, and statistical analysis was performed using GraphPad Prism 7.

SAXD experiments were performed to investigate repetitive structures in the turbid samples. 10 and 20 μM proteins were mixed with SUVs of 1–3 mM DMPC:DMPG (1:1) in HBS at ambient temperature and exposed at 25°C on the EMBL P12 BioSAXS beamline, DESY (Hamburg, Germany). A HBS buffer reference was subtracted from the data. Lipid samples without added protein did not produce Bragg peaks. The peak positions of momentum transfer, *s*, in all measured samples were used to calculate mean repeat distances, *d*, in proteolipid structures, using the equation

$$d=\frac{2\pi }{s},where\;s=\frac{4\pi sin\theta }{\lambda }$$

### Electron microscopy

For negatively stained EM, 740 μM DMPC:DMPG (1:1) SUVs were mixed with proteins using protein-to-lipid ratios of 1:58, 1:100, 1:200, and 1:500 and incubated at 22°C for 1 h. EM grids were then prepared, stained and imaged as described before [5,20,50].

### Neutron reflectometry

Supported lipid bilayers were prepared onto flat (5 Å RMS roughness tolerance) 80 mm × 50 mm ×15 mm Si-crystal blocks (Sil’tronix Silicon Technologies, Archamps, France). Samples were prepared from a chloroform-methanol stock of 1 mg/ml DMPC:DMPG (1:1). Using Langmuir-Blodgett and Langmuir-Schaefer techniques, the two membrane leaflets of the bilayers were deposited sequentially. The surface pressure was kept at a constant 30 mN m^-1^ during the deposition, as described previously [20,51,52]. All sample blocks were assembled into low-volume measurement flow cells, which were used for *in situ* exchange of solvent and injection of protein samples between reflectometric data collections [53].

Neutron reflectometric measurements for wt-P0ct were performed as described [20]. In short, the D17 neutron reflectometer at the Institut Laue-Langevin (Grenoble, France) was used for data collection at two incident angles (0.8° and 3.2°) [54]. All samples were kept at 30°C with HBS buffer as the liquid phase, prepared at a final concentration of 95% (v/v) deuterium oxide (D_2_O, Sigma-Aldrich) and in H_2_O. The deposited bilayers were characterized, before and after the injection of P0ct, at three different solvent contrasts, varying the volume ratio of D_2_O and H_2_O in to the sample cell: (1) 95% D_2_O, (2) Si-matched water (SMW; 38% (v/v) D_2_O, 62% (v/v) H_2_O), and (3) 100% H_2_O. A 0.5 μM P0ct solution was allowed to interact with the membrane for 3 h whilst monitoring reflectivity, until no further changes were observed. Any excess P0ct was washed out from the bulk solution by exchanging 20 cell volumes of solvent slowly through the sample cell. Fitting was performed using Motofit in Igor Pro 7 [55].

The scattering length densities of the phospholipids were calculated from volume fractions of the lipid components obtained from molecular dynamics simulations [56], and for the proteins, they were calculated from the sequences and amino acid volumes [57]. The P0ct scattering length density, assuming 90% labile hydrogen exchange, was 3.227, 2.324, and 1.722 x 10^−6^ Å^-2^ in 95%, 38%, and 0% D_2_O, respectively. The errors in the structural parameters for each sublayer were derived from the maximum acceptable variation in the fitted thickness and lipid volume fraction that allowed a fit to be maintained, subject to a constant molecular area constraint required to maintain a planar bilayer geometry.

Details of the analysis of supported lipid membrane structure [58] and interaction with soluble proteins [59] using time-of-flight neutron reflection have been described previously. The fraction of P0 in the lipid bilayers was determined by a simultaneous fit to all contrasts, taking into account the change in protein scattering length density with solvent contrast due to H/D exchange of protons on polar residues with the solvent.

For mutant comparison to wt-P0ct, NR data for wt-P0ct and D224Y were collected on the INTER neutron reflectometer at ISIS Neutron and Muon Source (Didcot, United Kingdom) at two incident angles (0.7° and 2.3°) [60] covering a total q-range from 0.01 to 0.34 Å^-1^, with a resolution of Δq/q = 0.03. The samples were prepared and handled as above.

### Modelling

A 3D model for the neuritogenic segment was made using PEP-FOLD [61]. The peptide was then subjected to MD simulations at +25°C in water, using YASARA [62], essentially as described [63].

## Supporting information

S1 Fig Protein yield. The purified protein amount from *E*. *coli* expression, shown as mg of protein obtained per 1 l of culture.
(JPG)

S2 Fig The folding of P0ct variants in TFE, detergents, and poorly binding membrane compositions. The folding of wt-P0ct and mutants was studied using SRCD spectropolarimetry in (a) 10% TFE, (b) 50% TFE, (c) 70% TFE, (d) 0.1% DPC, (e) 1% LDAO, (f) 1% OG, (g) DMPC, and (h) 9:1 DMPC:DMPG. The colour coding legend in panel (a) for each mutant trace also corresponds to all other traces in subsequent panels.
(JPG)

S3 Fig P0ct variant-induced turbidimetry and diffraction. (a) Turbidimetric analysis of 0.5 mM DMPC:DMPG (1:1) vesicles in the presence of 0–10 μM wt-P0ct and mutants. BSA was included as negative control. Error bars represent standard deviation. (b) Examples of Bragg peaks from the P0ct samples mixed with DMPC:DMPG (1:1) vesicles.
(JPG)

S4 Fig EM analysis of P0ct D224Y. Negatively stained samples of DMPC:DMPG (1:1) vesicles mixed with P0ct D224Y at (a) 1:100, (b) 1:200, and (c) 1:500 P/L ratios all display multilayered lipid structures.
(JPG)

S5 Fig NR data of wt-P0ct and D224Y. NR data for DMPC:DMPG (1:1)-bound wt-P0ct and D224Y. The data have been offset for clarity. Solvent contrasts are indicated for each trace on their right hand side. The D224Y H_2_O data is incomplete as reflectivity was collected at only one measurement angle (0.7°). The error bars denote standard deviation.
(JPG)

S1 Table DLS parameters. (DOCX)

S2 Table SAXS parameters. (DOCX)

S3 Table DSC fitting results. (DOCX)

S1 Supplementary Data Raw numerical data used for preparing all graphs. (ZIP)
