# Supplementary figures and images for: Neuropathy-related mutations alter the membrane binding properties of the human myelin protein P0 cytoplasmic tail
Source: PLoS One. 2019 Jun 7;14(6):e0216833. doi: 10.1371/journal.pone.0216833 (PMC6555526; doi:10.1371/journal.pone.0216833)

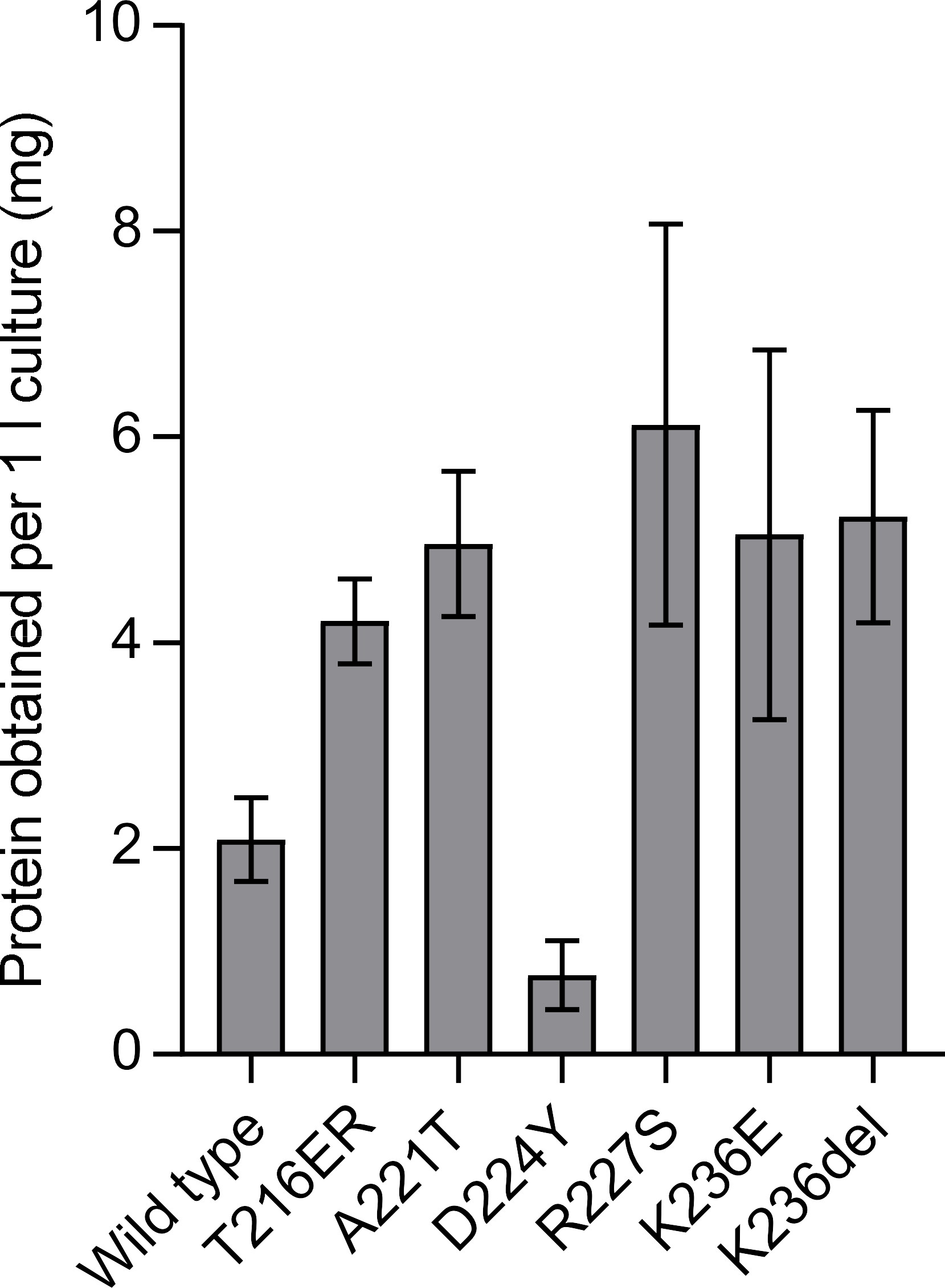

Supplement: S1 Fig — The purified protein amount from E. coli expression, shown as mg of protein obtained per 1 l of culture. (JPG) [file pone.0216833.s001.jpg]

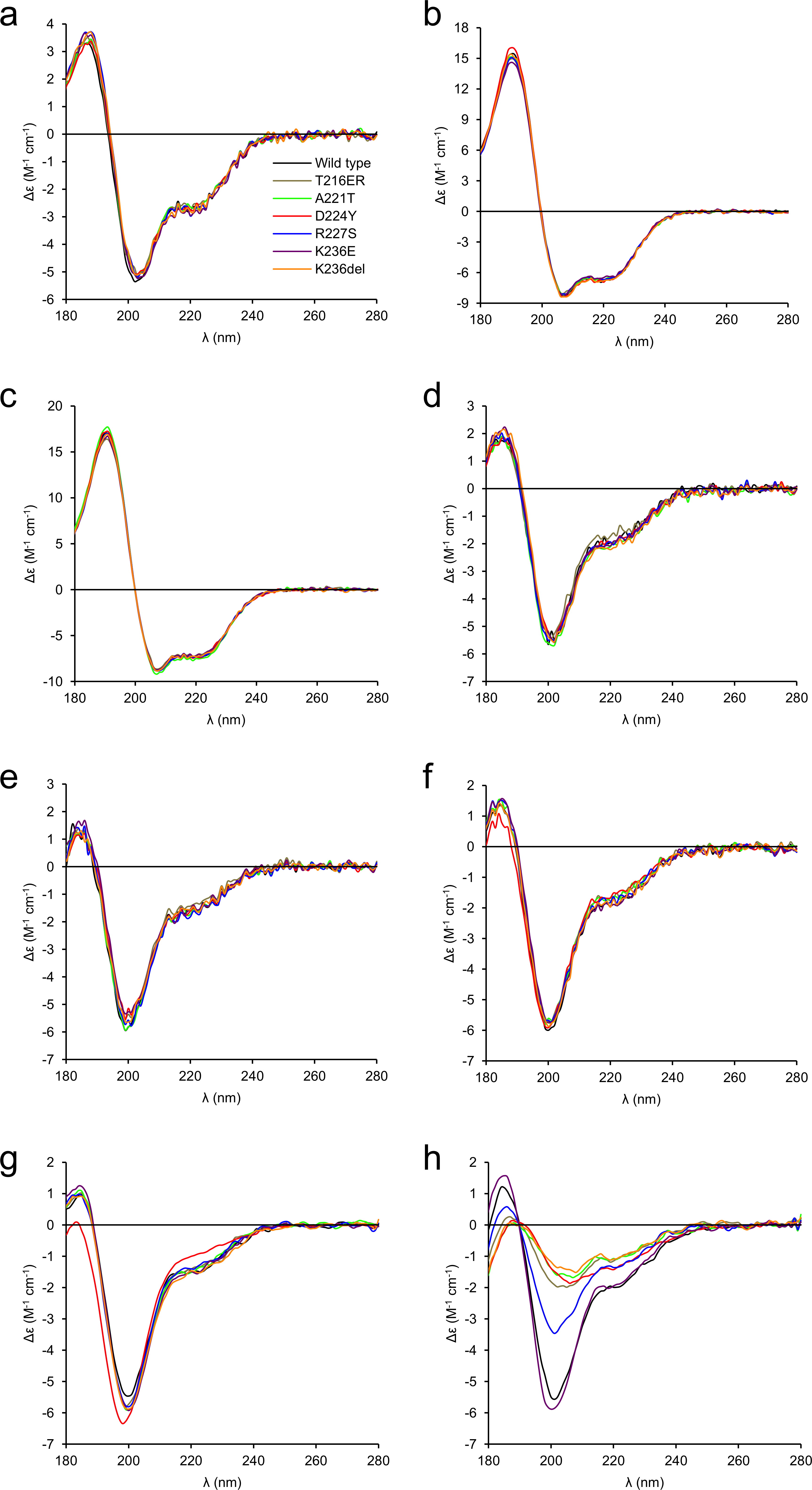

Supplement: S2 Fig — The folding of wt-P0ct and mutants was studied using SRCD spectropolarimetry in (a) 10% TFE, (b) 50% TFE, (c) 70% TFE, (d) 0.1% DPC, (e) 1% LDAO, (f) 1% OG, (g) DMPC, and (h) 9:1 DMPC:DMPG. The colour coding legend in panel (a) for each mutant trace also corresponds to all other traces in subsequent panels. (JPG) [file pone.0216833.s002.jpg]

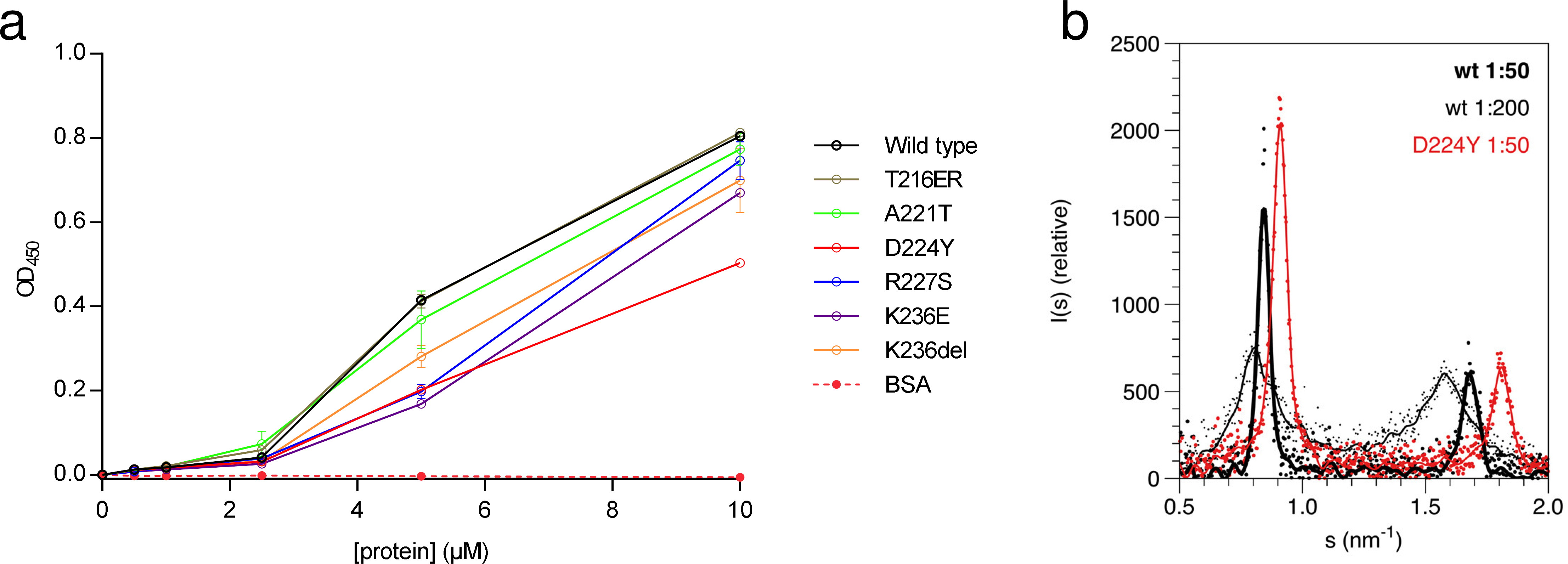

Supplement: S3 Fig — (a) Turbidimetric analysis of 0.5 mM DMPC:DMPG (1:1) vesicles in the presence of 0–10 μM wt-P0ct and mutants. BSA was included as negative control. Error bars represent standard deviation. (b) Examples of Bragg peaks from the P0ct samples mixed with DMPC:DMPG (1:1) vesicles. (JPG) [file pone.0216833.s003.jpg]

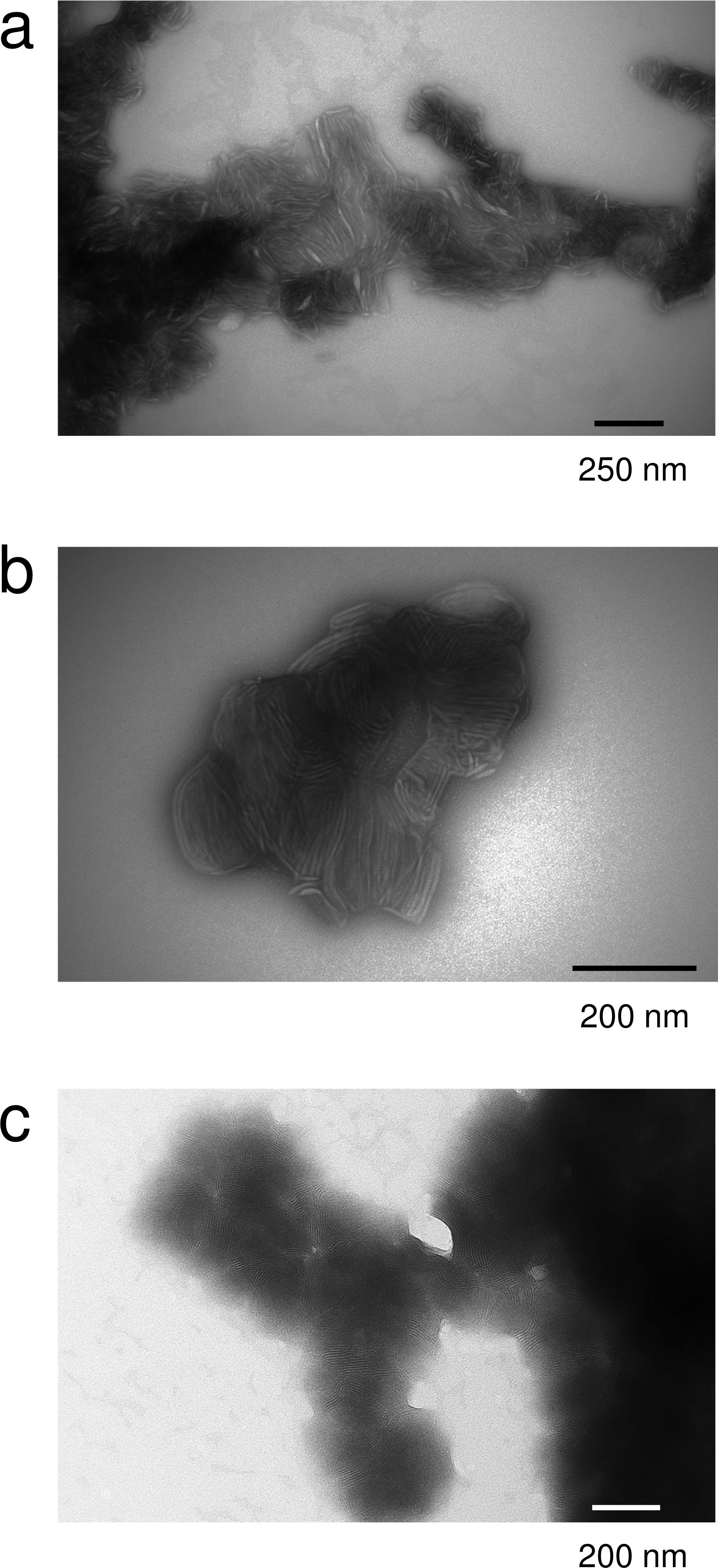

Supplement: S4 Fig — Negatively stained samples of DMPC:DMPG (1:1) vesicles mixed with P0ct D224Y at (a) 1:100, (b) 1:200, and (c) 1:500 P/L ratios all display multilayered lipid structures. (JPG) [file pone.0216833.s004.jpg]

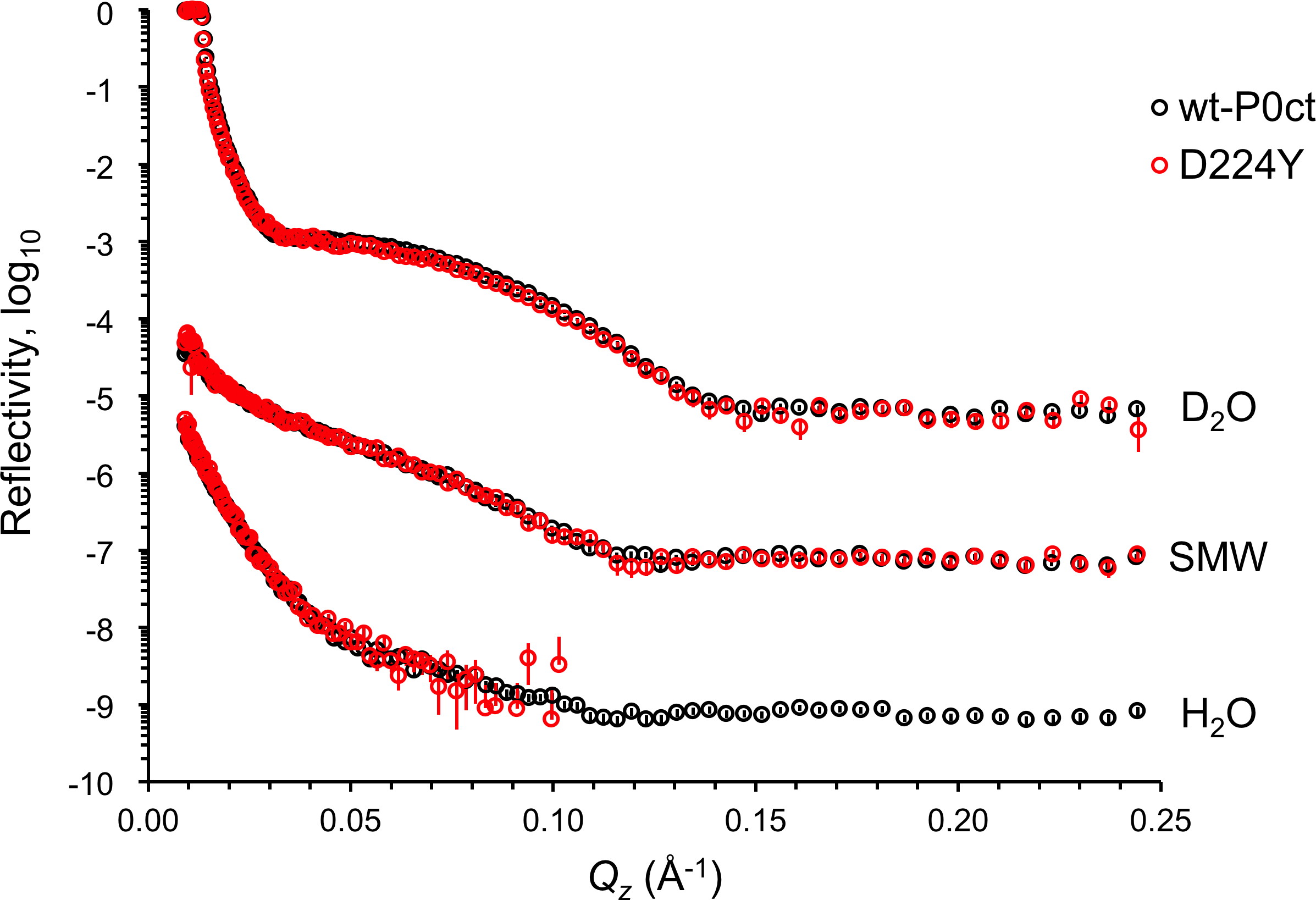

Supplement: S5 Fig — NR data for DMPC:DMPG (1:1)-bound wt-P0ct and D224Y. The data have been offset for clarity. Solvent contrasts are indicated for each trace on their right hand side. The D224Y H2O data is incomplete as reflectivity was collected at only one measurement angle (0.7°). The error bars denote standard deviation. (JPG) [file pone.0216833.s005.jpg]
