# Supplementary material for: Neuropathy-related mutations alter the membrane binding properties of the human myelin protein P0 cytoplasmic tail
Source: PLoS One. 2019 Jun 7;14(6):e0216833. doi: 10.1371/journal.pone.0216833 (PMC6555526; doi:10.1371/journal.pone.0216833)
Supplement: S1 Table — (DOCX) [file pone.0216833.s006.docx]

**Supplementary Table S1.** DLS parameters.

| Protein variant | wt-P0ct | T216ER | A221T | D224Y | R227S | K236E | K236del |
| --- | --- | --- | --- | --- | --- | --- | --- |
| Sample buffer* | HBS | HBS | HBS | TBS | HBS | HBS | HBS |
| *R*_h_ (nm) | 2.96 | 2.87 | 2.72 | 2.26 | 2.93 | 2.88 | 2.80 |

*HBS, 20 mM HEPES, 150 mM NaCl, pH 7.5; TBS, 20 mM Tris-HCl, 300 mM NaCl, pH 8.5.
