# Supplementary material for: Neuropathy-related mutations alter the membrane binding properties of the human myelin protein P0 cytoplasmic tail
Source: PLoS One. 2019 Jun 7;14(6):e0216833. doi: 10.1371/journal.pone.0216833 (PMC6555526; doi:10.1371/journal.pone.0216833)
Supplement: S2 Table — (DOCX) [file pone.0216833.s007.docx]

**Supplementary Table S2.** SAXS parameters.

| **Data collection parameters** | | | | | | | |
| --- | --- | --- | --- | --- | --- | --- | --- |
| Instrument | P12, PETRAIII, DESY | | | | | | |
| Wavelength (nm) | 0.124 | | | | | | |
| Angular range (nm^-1^) | 0.0403 - 7.3195 | | | | | | |
| Exposure time (s) | 0.045 | | | | | | |
| Measurement temperature (°C) | 10 | | | | | | |
| Protein variant | wt-P0ct | T216ER | A221T | D224Y | R227S | K236E | K236del |
| Concentration range (mg ml^-1^) | 0.3 - 1.2 | 1.0 - 3.8 | 2.0 - 8.0 | 0.5 - 2.1 | 1.7 - 6.8 | 3.5 - 12.9 | 2.3 - 9.3 |
| Sample buffer* | HBS | HBS | HBS | TBS | HBS | HBS | HBS |
| **Structural parameters** | |  |  |  |  |  |  |
| *I*_0_ (relative) [from p(r)] | 58.90 | 64.72 | 59.06 | 58.27 | 56.14 | 62.73 | 58.17 |
| *R*_g_ (nm) [from p(r) ] | 2.57 | 2.50 | 2.40 | 2.73 | 2.42 | 2.41 | 2.41 |
| *I*_0_ (relative) [from Guinier] | 58.25 | 63.87 | 58.35 | 57.38 | 55.21 | 61.61 | 57.30 |
| *R*_g_ (nm) [from Guinier] | 2.39 | 2.33 | 2.26 | 2.43 | 2.25 | 2.23 | 2.23 |
| D_max_ (nm) [from GNOM] | 9.59 | 9.21 | 9.59 | 11.57 | 8.96 | 10.34 | 10.69 |
| **Molecular mass determination** | | | | | | | |
| Molecular mass M_r_ (kDa) [from *I*_0_ using p(r)] | 7.87 | 8.65 | 7.89 | 7.79 | 7.50 | 8.38 | 7.78 |
| Molecular mass M_r_ (kDa) [from *I*_0_ using Guinier] | 7.79 | 8.54 | 7.80 | 7.67 | 7.38 | 8.24 | 7.66 |
| Theoretical M_r_ from sequence (kDa) | 7.99 | 8.17 | 8.02 | 8.04 | 7.92 | 7.99 | 7.86 |
| **Software** | | | | | | | |
| Primary data reduction & processing | PRIMUS | | | | | | |

*HBS, 20 mM HEPES, 150 mM NaCl, pH 7.5; TBS, 20 mM Tris-HCl, 300 mM NaCl, pH 8.5.
