# Supplementary material for: Neuropathy-related mutations alter the membrane binding properties of the human myelin protein P0 cytoplasmic tail
Source: PLoS One. 2019 Jun 7;14(6):e0216833. doi: 10.1371/journal.pone.0216833 (PMC6555526; doi:10.1371/journal.pone.0216833)
Supplement: S3 Table — (DOCX) [file pone.0216833.s008.docx]

**Supplementary Table S3.** DSC fitting results for the main phase transition peak. Phase transition temperatures for each sample were obtained through Lorentzian fitting, and the standard deviations come from two independent measurements for each sample.

| Sample | Lipids alone | wt-P0ct | T216ER | A221T | D224Y | R227S | K236E | K236del |
| --- | --- | --- | --- | --- | --- | --- | --- | --- |
| Main phase transition (°C) | 23.85±0.04 | 22.98±0.06 | 23.10±0.16 | 23.14±0.12 | 22.81±0.09 | 23.04±0.22 | 22.76±0.19 | 22.83±0.12 |
| Difference to lipids alone | - | -0.87 | -0.75 | -0.71 | -1.04 | -0.81 | -1.09 | -1.02 |
| Difference to wt-P0ct | +0.87 | - | +0.12 | +0.16 | -0.17 | +0.06 | -0.22 | -0.15 |
